# Genomics of Three New Bacteriophages Useful in the Biocontrol of *Salmonella*

**DOI:** 10.3389/fmicb.2016.00545

**Published:** 2016-04-20

**Authors:** Carlota Bardina, Joan Colom, Denis A. Spricigo, Jennifer Otero, Miquel Sánchez-Osuna, Pilar Cortés, Montserrat Llagostera

**Affiliations:** Departament de Genètica i de Microbiologia, Molecular Microbiology, Universitat Autònoma de BarcelonaBarcelona, Spain

**Keywords:** *Salmonella*, bacteriophage, genomics, chromosomal ends, *Myoviridae*, *Podoviridae*

## Abstract

Non-typhoid *Salmonella* is the principal pathogen related to food-borne diseases throughout the world. Widespread antibiotic resistance has adversely affected human health and has encouraged the search for alternative antimicrobial agents. The advances in bacteriophage therapy highlight their use in controlling a broad spectrum of food-borne pathogens. One requirement for the use of bacteriophages as antibacterials is the characterization of their genomes. In this work, complete genome sequencing and molecular analyses were carried out for three new virulent *Salmonella*-specific bacteriophages (UAB_Phi20, UAB_Phi78, and UAB_Phi87) able to infect a broad range of *Salmonella* strains. Sequence analysis of the genomes of UAB_Phi20, UAB_Phi78, and UAB_Phi87 bacteriophages did not evidence the presence of known virulence-associated and antibiotic resistance genes, and potential immunoreactive food allergens. The UAB_Phi20 genome comprised 41,809 base pairs with 80 open reading frames (ORFs); 24 of them with assigned function. Genome sequence showed a high homology of UAB_Phi20 with *Salmonella* bacteriophage P22 and other *P22likeviruses genus* of the *Podoviridae* family, including ST64T and ST104. The DNA of UAB_Phi78 contained 44,110 bp including direct terminal repeats (DTR) of 179 bp and 58 putative ORFs were predicted and 20 were assigned function. This bacteriophage was assigned to the *SP6likeviruses* genus of the *Podoviridae* family based on its high similarity not only with SP6 but also with the K1-5, K1E, and K1F bacteriophages, all of which infect *Escherichia coli.* The UAB_Phi87 genome sequence consisted of 87,669 bp with terminal direct repeats of 608 bp; although 148 ORFs were identified, putative functions could be assigned to only 29 of them. Sequence comparisons revealed the mosaic structure of UAB_Phi87 and its high similarity with bacteriophages Felix O1 and wV8 of *E. coli* with respect to genetic content and functional organization. Phylogenetic analysis of large terminase subunits confirms their packaging strategies and grouping to the different phage genus type. All these studies are necessary for the development and the use of an efficient cocktail with commercial applications in bacteriophage therapy against *Salmonella*.

## Introduction

Non-typhoid *Salmonella* is the leading reported pathogen related to food-borne diseases, both in the European Union (EU) (European Food Safety Authority and European Centre for Disease Prevention Control, 2014) and in the USA (CDC, 2011). Salmonellosis in humans is often related to the ingestion of contaminated animal products (poultry, swine, beef, etc.) or of fruits and vegetables contaminated by animal waste (European Food Safety Authority and European Centre for Disease Prevention Control, 2014), consistent with the prevalence of certain serovars of *Salmonella enterica* in farm animals (e.g., Typhimurium and Enteritidis). The widespread antibiotic resistance in bacteria from various sources has had adverse effects on human health and has therefore encouraged the search for alternative antimicrobial agents (Endersen et al., 2014).

The natural biotherapeutic potential of bacteriophages is well recognized. Since 2006, different bacteriophage products have been assayed for use as therapeutics and food safety agents (Sulakvelidze, 2011). Bacteriophages and their derivatives are promising resources for use at each stage of the farm-to-fork. Recently, it has been reviewed their use for controlling of several major and emerging food-borne pathogens in both preharvest (farm animals) and postharvest (meat, fresh, and packaged foods) environments (Goodridge and Bisha, 2011). These studies reinforce the commercially exploiting of bacteriophages to diminish the economic weight of microbial contamination in foods and food processing environments.

To date, there is no evidence that bacteriophages exhibit harmful effects on humans or animals (Abedon et al., 2011). They are the most abundant entities and are present in all environments where a suitable host is found due to their high degree of host specificity (Kropinski et al., 2007). Nowadays, a security measure in the use of bacteriophages as antibacterials is that they must undergo whole-genome sequencing to ensure that the genome is free of genes encoding known bacterial virulence factors and potential immunoreactive allergens. Moreover, sequencing helps to understand the multiplicative cycle of bacteriophages at molecular level, and also other important biological traits. With this aim, the present work reports the sequencing and detailed analysis of the genomes of three *Salmonella*-specific bacteriophages (UAB_Phi20, UAB_Phi78, and UAB_Phi87) and the identification of the type of their genome ends. All three bacteriophages are able to infect not only a broad range of different strains of *S*. Typhimurium and *S.* Enteritidis serovars but also strains of the serovars Virchow, Hadar, and Infantis. They were previously selected from a collection of 55 bacteriophages isolated in poultry and pig feces obtained at different farms in Spain (Cortés et al., 2015). These bacteriophages are efficient against *S*. Typhimurium, both in poultry (Bardina et al., 2012; Colom et al., 2015) and in different food matrices (Spricigo et al., 2013).

## Materials AND methods

### Bacteriophages

The three bacteriophages studied in this work, UAB_Phi20, UAB_Phi78, and UAB_Phi87, belong to a collection of 55 bacteriophages previously obtained from 189 chicken cloacae and pig rectal swabs collected from farms in different geographical areas of Spain between 2007 and 2009 (Cortés et al., 2015).

### Bacteriophage DNA extraction

High-titer (10^11^–10^12^ pfu/ml in MgSO_4_ 10 mM) lysates were obtained from each bacteriophage propagated in *S.* Typhimurium LB5000 strain (SGSC181; University of Calgary) and by ultracentrifugation at 51,000 × g for 2 h (Optima^*TM*^ L-80; Beckman, CA, USA) (Sambrook et al., 1989). Bacteriophage DNA was isolated using a phenol-chloroform method (Sambrook et al., 1989) with slight modifications. Phage suspensions were treated with DNase I (80 U/ml; Roche Diagnostics GmbH, Germany) and RNase I (80 μg/ml; Roche Diagnostics GmbH, Germany) at 37°C for 2 h. Following the addition of 0.5% sodium dodecyl sulfate (SDS, Sigma-Aldrich, St. Louis, MO, USA) and 200 μg proteinase K (Roche Diagnostics GmbH, Germany)/ml, they were incubated at 56°C for 2 h. Phage DNA was then extracted using phenol:chloroform and precipitated with ethanol. DNA integrity was checked by using a 0.7% agarose gel electrophoresis stained with Red Safe 1X (Intron Biotechnology; Seongnam-Si, Korea); the concentration was determined in a NanoDrop ND 1000 instrument (Thermo Scientific, DE, USA).

### Bacteriophage DNA sequencing and genomic analysis

The genomes of UAB_Phi20 and UAB_Phi78 were sequenced using the shotgun-full sequencing strategy. The UAB_Phi87 genome was sequenced using the Roche GS FLX system. All sequencing and sequence assembly procedures were done at Sistemas Genómicos (Valencia, Spain).

DNA sequences were analyzed using the software package DNAStar (DNAStar Inc.) and the online databases: http://www.ncbi.nlm.nih, http://www.ebi.ac.uk/, and http://cmr.jcvi.org. The whole-genome sequences of bacteriophages UAB_Phi20, UAB_Phi78, and UAB_Phi87 were deposited at GenBank under accession numbers GQ422450, GU595417, and JN225449, respectively. Possible open reading frames (ORFs) were predicted using the ORF Finder program (http://www.ncbi.nlm.nih.gov/gorf/gorf.html). ORFs > 25 amino acids in length were further analyzed. Putative functions of ORFs were identified using the alignment search tools (BLASTP, BLASTX, and BLASTN search) of the National Center for Biotechnology Information (NCBI). ATG, GTG, and TTG were considered as start codons and TAA, TGA, and TAG as stop codons. Potential promoter regions and transcription terminators were predicted using the Softberry programs BProm (http://linux1.softberry.com/berry.phtml), FindTerm (Solovyev and Salamov, 2011), and TransTerm (Ermolaeva et al., 2000). The presence of a putative Shine-Dalgarno sequence (ribosome binding site, RBS) was confirmed based on its similarity to the *Escherichia coli* consensus sequence GGAGGT (Shine and Dalgarno, 1974). The tRNAscan-SE 1.21 program was used to search putative tRNAs (Lowe and Eddy, 1997). BLASTX and BLASTP were used to search for similarities with proteins in the database (Altschul et al., 1990). MAUVE (Darling et al., 2010) or ClustalW2 (McWilliam et al., 2013) were used for genome comparisons at the nucleotide level based on the genomic sequences available at NCBI (www.ncbi.nlm.nih.gov). Comparisons at the proteomic level were made using CoreGenes (Turner et al., 2013). Phylogenetic analysis of phage large terminase subunit sequences was performed by using the ClustalW program in MEGA6 (Tamura et al., 2013). The tree based on neighbor-joining method was generated from a multiple alignment (gap opening penalty, 10; gap extension penalty, 1; and gap separation distance, 0). In order to obtain the tree, the parameters were set as following: (i) the model/method was set as number of differences; (ii) gaps/missing data treatment was established as complete deletion, and (iii) the random number generator seed and bootstrap trails were set at 111 and 1000, respectively (Casjens et al., 2005). Finally, the condensed tree was displayed with a bootstrap cut-off value of 70%.

### Determination of the bacteriophage genome ends

To identify potential *cos* ends, purified DNA of the three phages was digested with *Eco*RV restriction endonuclease (New England Biolabs, Hitchin, UK) at 37°C for 14 h. Two aliquots were then prepared. One was incubated at 60°C for 10 min to separate potentially ligated *cos* sites and immediately stored on ice. Restriction fragments length polymorphism patterns of heated and non heated aliquots were visualized by agarose gel electrophoresis (0.8%). Lambda bacteriophage DNA (Roche Diagnostics GmbH, Germany) with cohesive ends and treated with the same methodology served as a control.

On the other hand, the DNA from UAB_Phi20 was digested with *Eco*RI enzyme (New England Biolabs, Hitchin, UK) to detect an under-represented fragment as indicative of circularly permuted direct terminal repeats (DTR) in the chromosome ends. DNA of P22 bacteriophage was used as a control. Finally, to determine if the chromosome ends of UAB_Phi78 and UAB_Phi87 bacteriophages contain DTR, their DNA was treated with exonuclease *Bal*31, as described elsewhere (Klumpp et al., 2008). Briefly, 30 μg of bacteriophage DNA was treated with *Bal*31 nuclease (Takara; Saint-Germain-en-Laye, France) (0.5 units/μg) at 30°C for different incubation times. The reaction was stopped by the addition of 10 μl of EDTA (20 mM) followed by heating at 65°C for 5 min. DNA was purified using phenol-chloroform extraction and ethanol precipitation (Sambrook et al., 1989). Purified DNA (1 μg) was digested with *Hin*dIII (UAB_Phi78) and *Spe*I (UAB_Phi87) restriction enzymes (New England Biolabs, Hitchin, UK) and analyzed by agarose gel electrophoresis (1%). Those fragments that disappeared were newly isolated and purified (GE Healthcare Ltd., UK). *In silico* restriction of UAB_Phi78 and UAB_Phi87 with the adequate cutting sites were performed in order to identify the sequence of the disappeared fragments. In attention to these results, different primers were designed for sequencing the recovered and purified fragments. Finally, the sequences of DTR for both phages were confirmed by sequencing. To do this, the phage genomes were used as templates with primers that previously displayed drop-offs in the sequencing of the recovered fragments.

### Isolation of UAB_Phi20 lysogens

The possible lysogens present in the clear plaques of bacteriophage UAB_Phi20 on *S*. Typhimurium ATCC 14028 were picked from 10 plaques and streaked on green plates (Chan et al., 1972). Forty colonies were selected from these plates and streaked on green plates several times until they did not show dark green color. Overnight cultures in LB liquid medium of each colony were obtained and subcultured until an optical density at 550 nm (OD_550_) of 1.0 was reached. Following, 0.5 μg/ml of mitomycin C (Sigma, St. Louis, MO) was added to the cultures and incubated at 37°C for 2 h. At that time, cultures were centrifuged and filtrated. A spotting assay of the supernatants with *S*. Typhimurium ATCC 14028 was conducted to ascertain the presence of induced UAB_Phi20. Similarly, a spotting assay of supernatants of overnight cultures was done.

## Results and discussion

The adsorption kinetics and the lytic cycle of bacteriophages UAB_Phi20, UAB_Phi78, and UAB_Phi87 used as a cocktail in therapy strategies against *S.* Typhimurium (Bardina et al., 2012; Spricigo et al., 2013; Colom et al., 2015) were previously characterized. They exhibited similar adsorption constant (K) ranging between 1.1 × 10^−9^ and 1.2 × 10^−9^ ml cfu^−1^ min^−1^ and the timing of the latent period of bacteriophages UAB_Phi20, UAB_Phi78, and UAB_Phi87 was 46.0, 26.7, and 58.0 min, respectively (Bardina, 2011; Spricigo, 2011). The burst sizes of UAB_Phi20 and UAB_Phi78 were similar (95.0 and 87.7 pfu/cfu, respectively) while that of UAB_Phi87 was 55 pfu/cfu (Bardina, 2011; Spricigo, 2011). In addition, they were previously characterized with respect to broad host range, restriction patterns, RAPD profiles, morphology, genome size and lytic activity *in vitro* (Bardina et al., 2012; Cortés et al., 2015).

In this study, we report the whole genome sequencing and some traits of their biology at molecular level. *In silico* analyses of bacteriophage genomes did not show any similarities neither to known virulence-associated genes nor to any antibiotic resistance genes or potential immunoreactive food allergens (FARRP, 2011). It must be noted that a high percentage of hypothetical proteins were found in their genomes. This agrees with the reported for all sequenced bacteriophages which has been widely commented by the scientific community (Klumpp et al., 2013). Therefore, the identification of their function is a challenge that must be addressed for increasing the knowledge of the bacteriophages and the level of security of their applications. In this regard, it must be considered that none of hypothetical proteins showed significant similarity to known or hypothetical factors involved in bacterial pathogenicity. Therefore, it is unlikely that they have a role in bacterial virulence. Additionally, in our reported *in vivo* experiments (Bardina et al., 2012), we inoculated the phage cocktail, with and without their host, and no harmful signs were observed in animals. In attention to the above indicated, and given the large amount of information available in bacterial gene databases, the three phages studied are safe with respect to our current knowledge.

### Genome analysis of UAB_Phi20

**Figure 1:**
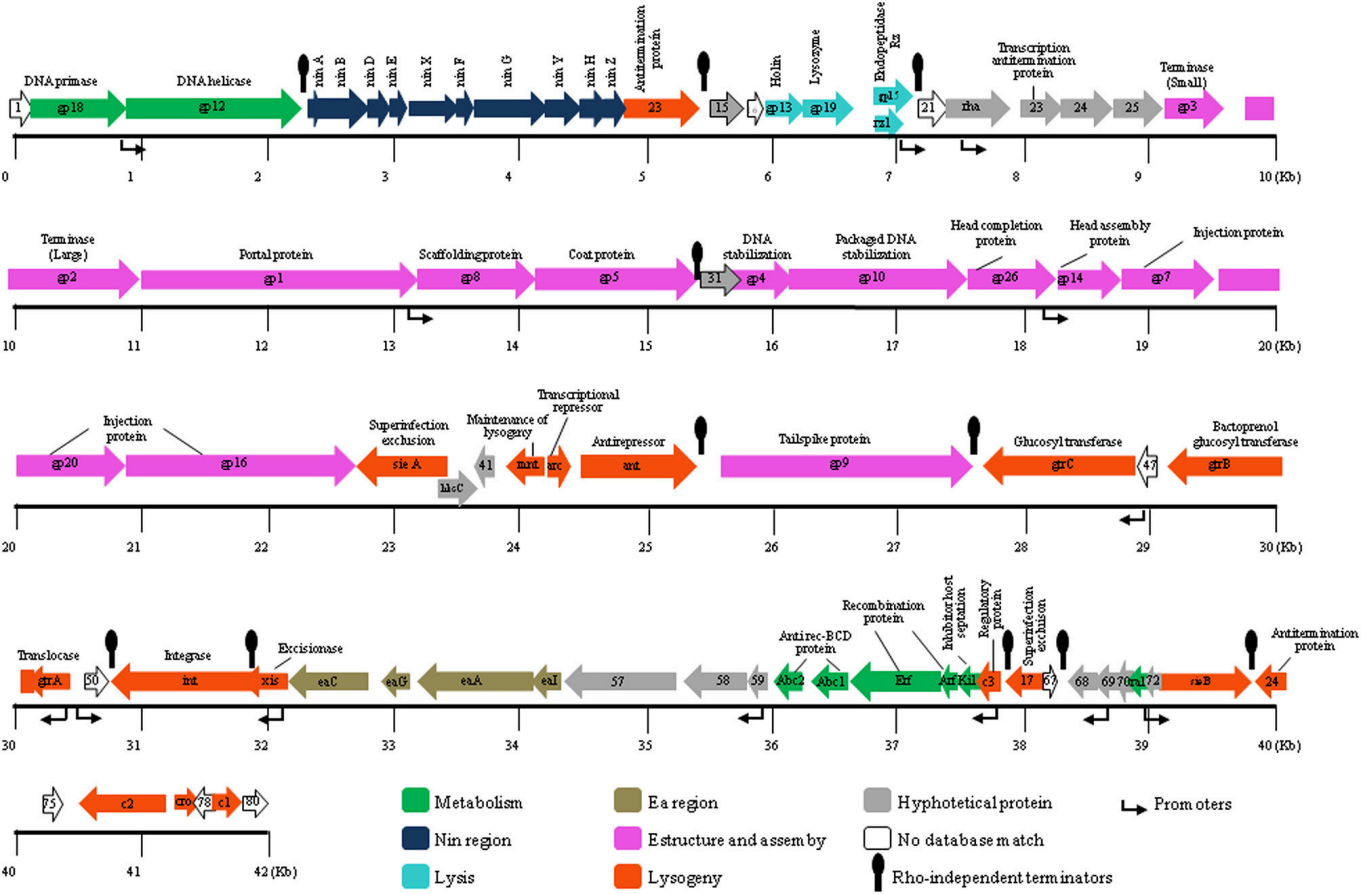
**Genomic structure of bacteriophage UAB_Phi20 including the Rho-indepedent terminators**. Arrows represent genes, and the different colors identify the functional category into which the homologous genes were classified. Gene functions are indicated where they are known. The color code for gene function is provided at the bottom of the figure. ORFs are numbered consecutively from left to right as described in Table 1, and are indicated by arrows pointing to the direction of transcription.

The genome of UAB_Phi20 consisted of linear double-stranded DNA (ds DNA), 41,809 base pairs (bp) in length and with an overall genomic guanine plus cytosine (G+C) content of 47.2% which is slightly lower than its host (52.2%). ORF Finder revealed 80 possible ORFs. The annotation and organization of the UAB_Phi20 genome are provided in Table 1. Given the high level of genome compaction, many of the promoters identified in UAB_Phi20 overlapped in their coding regions. Therefore, potential promoters were sought using the BPROM program (Softberry), limiting the search to a maximum distance of 100 bp relative to the start of the potential UAB_Phi20 phage genes. All 12 hypothetical promoters thus identified (Table S1) had a highly conserved −10 consensus sequence (TATAAT), while in the −35 box (TTGACA) only the second T and the G were strongly conserved. In addition, there were 11 putative Rho-independent terminators (Figure 1). ATG was the start codon in all ORFs except gene *p80*, in which TTG was the start codon. The three stop codons were present in different proportions, with TAA as the most common (56.3% of the genes), followed by TGA and TAG (in 36.2 and 7.5% of the ORFs, respectively). The RBS Finder (Glimmer) program revealed partial conservation of the ribosome-binding sites (RBS) of bacteriophage UAB_Phi20 with respect to the Shine-Dalgarno consensus sequence (AGGAGG). Interestingly, the distance of this sequence from the translation initiation site was not conserved in all genes but instead ranged from only 7 to 40 bp.

**Table 1:** **Features of bacteriophage UAB_Phi20 genome, ORFs, gene products, and functional assignments**.

**ORF**	**Gene**	**Position (nt)**		**Strand**	**No of amino acids**	**Predictive function**	**Closest hit (Accession number)**	**% Amino acid identity**	**Best *e*-value**
		**From**	**To**						
1		27	173	+	48	Unknown	Hypothetical protein P22gp50(NP_059609.1)	100	9.E-25
2	*gp18*	166	981	+	271	DNA replication (primase)	Hypothetical protein P22gp51 (NP_059610.1)	100	0
3	*gp12*	978	2354	+	458	DNA replication (helicase)	Hypothetical protein P22gp52 (NP_059611.1)	100	0
4	*ninA*	2351	2431	+	26	Unknown	Nin A [Enterobacteria phage P22](YP_063725.1)	100	5.E-20
5	*ninB*	2428	2865	+	145	Unknown	NinB [Enterobacteria phage P22] (NP_059612.1)	99	6.E-101
6	*ninD*	2862	3035	+	57	Unknown	Nin D [Enterobacteria phage P22] (YP_063726.1)	100	7.E-35
7	*ninE*	3002	3178	+	58	Unknown	Nin E [Enterobacteria phage P22] (NP_059614.1)	100	6.E-34
8	*ninX*	3175	3513	+	112	Unknown	Nin X [Enterobacteria phage P22] (NP_059615.1)	100	1.E-77
9	*ninF*	3506	3682	+	58	Unknown	Nin F [Enterobacteria phage P22] (YP_063727.1)	100	2.E-33
10	*ninG*	3672	4286	+	203	Unknown	Nin G [Enterobacteria phage P22] (YP_063728.1)	100	6.E-145
11	*ninY*	4283	4507	+	74	Unknown	Nin Y [Enterobacteria phage P22] (NP_059618.1)	100	5.E-48
12	*ninH*	4504	4707	+	67	Unknown	Nin H [Enterobacteria phage P22] (NP_059619.1)	100	3.E-41
13	*ninZ*	4688	4867	+	59	Unknown	Nin Z [Enterobacteria phage P22] (YP_063729.1)	100	3.E-34
14	*23*	4864	5487	+	207	Transcription antitermination protein	Gp63 [Enterobacteria phage P22] (YP_063730.1)	100	9.E-153
15		5577	5786	+	69	Unknown	Hypothetical protein ε34gp63 (YP_002533523.1)	97	2.E-40
16		5809	5913	+	34	Unknown			
17	*gp13*	5922	6248	+	108	Holin	Holin [Enterobacteria phage P22] (NP_059621.1)	100	1.E-70
18	*gp19*	6229	6669	+	146	Lysozyme	Gp66 [Enterobacteria phage P22] (NP_059622.1)	100	1.E-101
19	*gp15*	6804	7103	+	99	Endopeptidase Rz	Hypothetical protein P22gp67 (NP_059623.2)	100	2.E-64
20	*Rz1*	6838	7050	+	70	Lipoprotein Rz1 precursor	Hypothetical protein P22gp68 (YP_063732.1)	100	1.E-34
21		7144	7329	+	61	Unknown			
22	*Rha*	7322	7858	+	178	Unknown	Rha [Enterobacteria phage P22] (NP_059624.1)	100	9.E-130
23		7940	8296	+	118	Unknown	Hypothetical protein SE1gp48 (YP_002455884)	100	7.E-78
24		8300	8689	+	129	Unknown	Hypothetical protein ST64Tp49 (NP_720323.1)	100	8.E-92
25		8689	9093	+	134	Unknown	Hypothetical protein ST64Tp50 (NP_720324.1)	100	2.E-90
26	*gp3*	9097	9585	+	162	Terminase (small subunit)	ST64Tp51 (NP_720325.1)	100	3.E-116
27	*gp2*	9737	11062	+	441	Terminase (large subunit)	Gp2 [Enterobacteria phage ST104] (YP_006405)	99	0
28	*gp1*	11062	13239	+	725	Portal protein	Gp1 [*Salmonella enterica* bacteriophage SE1] (YP_002455889.1)	100	0
29	*gp8*	13253	14164	+	303	Scaffolding protein	Gp8 [Enterobacteria phage P22] (YP_063736.1)	100	0
30	*gp5*	14164	15456	+	430	Coat protein	Coat protein [Enterobacteria phage ST64T] (NP_720329.1)	99	0
31		15495	15704	+	69	Unknown	Hypothetical protein P22gp06 (NP_059631.1)	100	5.E-40
32	*gp4*	15688	16188	+	166	DNA stabilization protein	Gp4[Enterobacteria phage P22] (NP_059632.1)	100	8.E-120
33	*gp10*	16148	17566	+	472	Packaged DNA stabilization protein	Head completion protein [Enterobacteria phage P22] (NP_059633.1)	100	0
34	*gp26*	17570	18271	+	233	Head completion protein	Gp26[Enterobacteria phage P22] (YP_063715.1)	100	5.E-165
35	*gp14*	18271	18726	+	151	Head assembly protein	Gp14 [Enterobacteria phage P22] (YP_063716.1)	100	6.E-109
36	*gp7*	18729	19418	+	229	Injection protein	Gp7 [Enterobacteria phage P22] (YP_063717.1)	100	2.E-154
37	*gp20*	19429	20844	+	471	Injection protein	Gp20 [Enterobacteria phage P22] (NP_059637.1)	100	0
38	*gp16*	20844	22673	+	609	Injection protein	Gp16 [Enterobacteria phage P22] (YP_063718.1)	100	0
39	*sieA*	23406	22696	−	236	Superinfection exclusion	SieA [Enterobacteria phage P22] (NP_059639.1)	99	7.E-110
40	*hkcC*	23221	23586	+	121	Unknown	hkcC [Bacteriophage HK620] (NP_112089.1)	100	5.E-85
41		23794	23600	−	64	Unknown	Hypothetical protein P22gp16 (NP_059640.1)	98	5.E-33
42	*mnt*	24130	23879	−	83	Maintenance of lysogeny	Mnt [Enterobacteria phage P22] (NP_059641.1)	100	2.E-53
43	*arc*	24158	24382	+	74	Transcriptional repressor	Repressor arc [Escherichia coli MS 16-3] (EFU59036.1)	99	8.E-46
44	*ant*	24451	25353	+	300	Antirepressor	Ant [Enterobacteria phage P22] (NP_059643.1)	100	0
45	*gp9*	25564	27567	+	667	Tailspike protein (Endorhamnosidase)	Tailspike protein [Enterobacteria phage P22] (AAF75060.1)	99	0
46	*gtrC*	28858	27626	−	410	O-antigen conversion; glucosyl transferase	GtrC [Enterobacteria phage P22] (YP_063719.1)	100	0
47		28879	28983	−	34	Unknown			
48	*gtrB*	30050	29073	−	325	O-antigen conversión; bactoprenol glucosyl transferase	GtrB [Enterobacteria phage ST64T] (NP_720276.1)	99	0
49	*gtrA*	30364	30002	−	120	O-antigen conversión; translocase (flipase)	GtrA [Enterobacteria phage P22] (NP_059583.1)	100	2.E-80
50		30538	30660	+	40	Unknown			
51	*int*	31876	30713	−	387	Integrase	Int [Enterobacteria phage P22] (NP_059584.1)	99	0
52	*xis*	32103	31753	−	116	Excisionase	Xis [Enterobacteria phage P22] (NP_059585.1)	100	3.E-79
53	*eaC*	32741	32106	−	211	Unknown	EaC [Enterobacteria phage P22] YP_063720.1	99	7.E-154
54	*eaG*	33021	32842	−	60	Unknown	EaG [Enterobacteria phage P22] (NP_059587.1)	100	4.E-34
55	*eaA*	34071	33118	−	317	Unknown	EaA [Enterobacteria phage P22] (NP_059588.1)	100	0
56	*eaI*	34269	34075	−	64	Unknown	EaI [Enterobacteria phage P22] (NP_059589.1)	100	3.E-36
57		35147	34266	−	293	Unknown	ORF8 [Enterobacteria phage ST104] (YP_006364.1)	91	1.E-116
58		35726	35217	−	169	Unknown	ORF9 [Enterobacteria phage ST104] (YP_006365.1)	100	2.E-118
59		35893	35723	−	56	Unknown	ORF10 [Enterobacteria phage ST104] (YP_006366.1)	100	7.E-33
60	*abc2*	36197	35904	−	97	Anti Rec-BCD protein	Abc2 [Enterobacteria phage ST104] (YP_006367.1)	100	4.E-63
61	*abc1*	36528	36244	−	94	Anti Rec-BCD protein	Abc1 [Enterobacteria phage ST104] (YP_006368.1)	100	3.E-62
62	*erf*	37235	36528	−	235	Recombination protein	ORF13 [Enterobacteria phage ST104] (YP_006369.1)	100	1.E-173
63	*arf*	37375	37232	−	47	Recombination protein	Arf [Enterobacteria phage P22] (NP_059597.1)	100	3.E-24
64	*kil*	37553	37365	−	62	Inhibitor of host septation	Kil [Enterobacteria phage P22] (NP_059598.1)	100	2.E-38
65	*c3*	37692	37534	−	52	Regulatory protein	C3 [Enterobacteria phage P22] (NP_059599.1)	100	2.E-29
66	*17*	38089	37778	−	103	Superinfection exclusion	Hypothetical protein P22gp40 (NP_059600.1)	100	9.E-70
67		38040	38159	+	39	Unknown			
68		38440	38237	−	67	Unknown	Hypothetical protein P22gp41 (CAA33649.1)	99	4.E-41
69		38676	38440	−	78	Unknown	Hypothetical protein P22gp42 (NP_059602.1)	100	2.E-47
70		38773	38648	−	41	Unknown	Hypothetical protein ST64Tp22 (NP_720296.1)	92	5.E-07
71	*ral*	38907	38713	−	64	Antirestriction protein	Ral [Enterobacteria phage P22] (NP_059603.1)	100	2.E-37
72		38977	38891	−	28	Unknown	Hypothetical protein lambdap47 (NP_040623.1)	100	4.E-27
73	*sieB*	38945	39700	+	251	Superinfection exclusion	SieB [Enterobacteria phage P22] (NP_059604.1)	100	3.E-138
74	*24*	40023	39721	−	100	Antitermination protein	Hypothetical protein P22gp46 (NP_059605.1)	100	3.E-65
75		40043	40255	+	70	Unknown			
76	*c2*	41027	40377	+	216	Prophage repressor	C2 [Enterobacteria phage P22] (NP_059606.1)	100	4.E-158
77	*cro*	41108	41293	+	61	Antirepressor	Cro [Enterobacteria phage P22] (NP_059607.1)	100	2.E-35
78		41354	41241	−	37	Unknown			
79	*c1*	41400	41678	+	92	Transcriptional activator	C1 [Enterobacteria phage P22] (NP_059608.1)	100	2.E-59
80		41668	41809	+	46	Unknown			

Gene numbers correspond with their predicted function, if known, followed by the nature of the evidence that supports the functional classification. Genes with no functional prediction, but with significant sequence similarity to genes in the NCBI database as determined by BLASTP are also listed.

The genomic annotation and the analysis of the genetic organization of phage UAB_Phi20 showed high homology with that of *Salmonella* bacteriophage P22 and other *P22likevirus*. Functions were assigned to 42 ORFs of the 80 identified (Table 1). In addition, 14 ORFs corresponded to *ea* and *nin* regions. Of the remaining 24 ORFs, 16 encoded proteins which showed similarity with hypothetical proteins already described, but their functions could not be determined, and 8 ORFs showed no similarity with any protein available in the databases. The proteins of UAB_Phi20 were classified into different functional groups (Figure 1).

The lysogeny group included proteins involved on the establishment of lysogeny, lysogenic conversion, immunity, the excisionase and the attP region. The establishment of lysogeny requires the activity of the integrase encoded by *int* gene. This protein showed an identity ≥98% compared to the counterparts in bacteriophages P22, ST64T, and ST104. A hypothetical *attP* site with a sequence similar to that described in P22 was also found between the genes *int* and *gtrA*. The products of the genes *c2, cro, c1*, and *c3* genes, which directly affect phage decision between lytic or lysogenic cycle, and those encoded by *mnt, arc*, and *ant* genes, which are involved in the control of the maintenance of lysogeny (Susskind and Botstein, 1978) showed a high similarity with those of P22. During lysogenic conversion, the lysogenization of bacterial cells with certain lambdoid bacteriophages produces a chemical change in the bacterial lipopolysaccharide O antigen such that the binding of other bacteriophages that recognize the same receptor is prevented (Kropinski et al., 2007). The UAB_Phi20 genes that are responsible for this function are *gtrC, gtrB*, and *gtrA*; all of their products showed ≥99% homology with their counterparts in bacteriophages P22, ST64T, and ST104. The genome of UAB_Phi20 also contains three genes (*17, sieA*, and *sieB*) encoding proteins involved in the exclusion of superinfection (immunity). These proteins were very similar to those of phage P22. Protein 17 participates in the release of exclusion by heterologous phages such as Fels-1, whereas SieB and SieA prevent infection by heteroimmune phages or superinfection by the own phage (Susskind and Botstein, 1978). In addition, the excisionase (*xis*) showed an identity of 100% compared to the corresponding protein in P22 and ST64T, whereas for ST104 the identity was ~97%.

The gene products involved in the DNA metabolism of UAB_Phi20 were identical to those of ST104 but had only ~70% identity with those of phage P22. This group of genes included *abc2* and *abc1*, encoding a protein with an anti-RecBCD function, and the hypothetical *erf* and *arf* genes, involved in the recombination and recircularization of phage DNA (Poteete et al., 1988). Genome replication by UAB_Phi20 requires two proteins similar to the helicase (Gp12) and primase (Gp18) proteins of P22 (Vander Byl and Kropinski, 2000).

A cluster of UAB_Phi20 genes involved in the bacterial lysis encoding holin (*gp13*), lysozyme (*gp19*), and two endopeptidases (*gp15* and *Rz1*) were identified. All these proteins were identical to those of phage P22. After the endopeptidases, the *orf21*, which may also play a role in bacterial lysis, was identified. However, as no gene homologous to *orf21* was found in the databases, neither its function nor its assignment to the lysis region could be confirmed.

Genes involved in structure and assembly could be divided into those encoding terminases, capsid, DNA injection, or tail proteins. The major part of proteins involved in these functions was similar to the respective proteins of phage P22 and presented a high identity with those encoded by bacteriophages ST104 and ST64T. For example, UAB_Phi20 tail-spike and the major capsid proteins were almost identical (≥99%) to the respective proteins of bacteriophages P22, ST104 and ST64T. Both the small and large terminases, encoded by *gp*3 and *gp2*, respectively, had an identity of ~100% with the genes of P22, ST104, and ST64T phages. In a recent work comparing 57 P22-like bacteriophages (Casjens and Thuman-Commike, 2011), terminases and capsid proteins were the most conserved, whereas the most divergent proteins were related to host recognition, such as tail and injection proteins. However, the high identity found by us for all these proteins indicates a low divergence and strong phylogenetic relationship between UAB_Phi20 and bacteriophages P22, ST104, and ST64T. In addition, the genome of UAB_Phi20 contained a unique site (*pac*) located within the sequence of the small subunit of terminase. *Pac* sequence (GAAGACTTATCTGAGGTCGTTA) differed by two bases from the corresponding sequence of P22 (Wu et al., 2002).

Besides regions above commented, other important feature of the UAB_Phi20 genome was the identification of *ea* and *nin* regions, which encoded a number of proteins of unknown function. Neither of these genes is essential for bacteriophage function, at least in *in vitro* cultures, but their presence and maintenance suggest that they confer a selective advantage for either the host or the bacteriophage itself when present in other environments (Hendrix, 2002).

Although the UAB_Phi20 genome contains all the elements for giving a lysogenic cycle, infected-*Salmonella* cultures by this bacteriophage were completely cleared and UAB_Phi20 plaques were also typically clear. Both observations suggested that this phage is virulent and unable to promote a lysogenic cycle. The possible reasons of this apparent contradiction were studied. First of all we considered that the hypothetical *attP* sequence of UAB_Phi20 is similar to that described in P22 bacteriophage. Therefore, bacteriophage UAB_Phi20 could integrate into the P22 site (*attB*) of the *Salmonella* chromosome (Smith-Mungo et al., 1994). However, we were unable to detect this by PCR amplification studies (data not shown). In addition, the possible C1 recognition motif (TTGN6TTGC) in the UAB_Phi20 genome was not identified neither at the region of the P_*RE*_ promoter nor in the vicinity of the gene encoding integrase and, as consequence, the repressor of the lytic cycle cannot be transcribed. However, these data did not discard that UAB_Phi20 had a very low frequency of lysogenization which could result in apparently clear plaques. To test this, the possible lysogens present in the clear plaques were picked and streaked on green plates. Afterwards, 40 colonies were selected from these plates and, for removing the possible bacteriophages coming from plaques, they were streaked on green plates until they did not show dark green color. If UAB_Phi20 had a low frequency of lysogenization, it would be expected that some of these colonies were stable lysogens. However, the treatment of liquid cultures of those colonies with mitomycin C did not yield bacteriophage production. In addition, no bacteriophages were detected in the supernatant of overnight cultures of these colonies. All these results evidenced that the bacteriophage UAB_Phi20 is unable to give rise a lysogenic cycle producing stable lysogens on this host.

**Figure 2:**
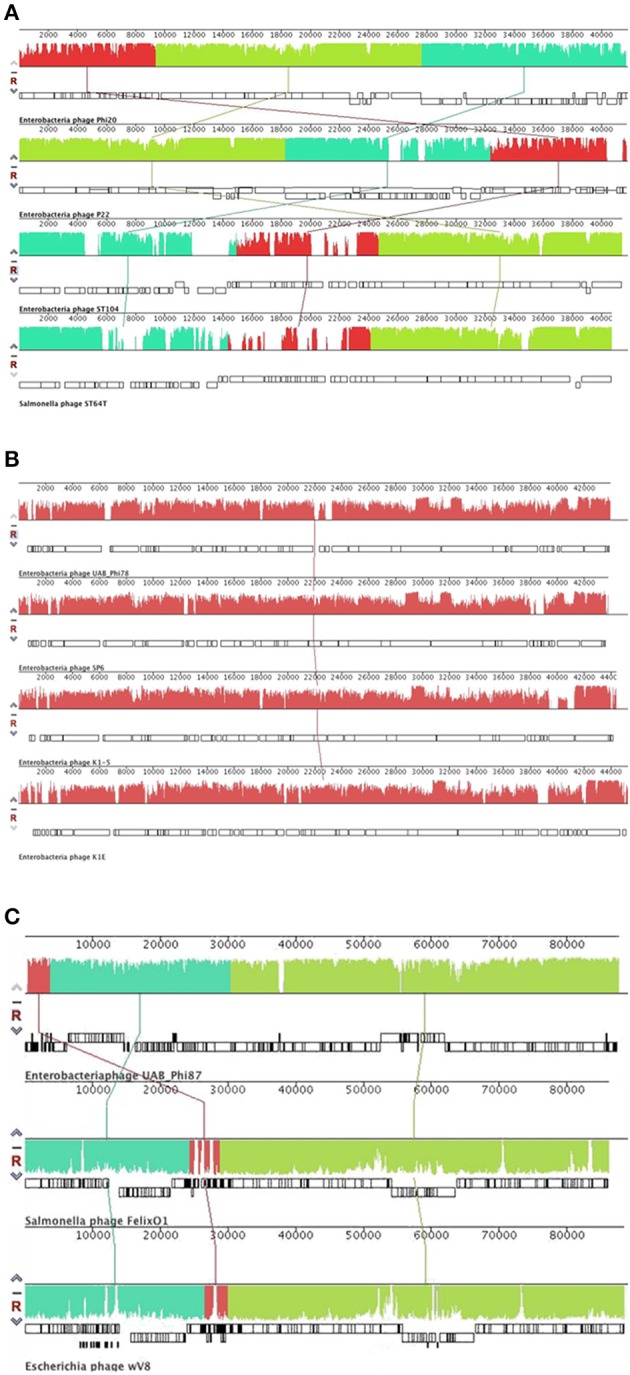
**Alignment of the UAB_Phi20 (A), UAB_Phi78 (B), and UAB_Phi87 (C) genomes and their counterparts belonging to the ***Podoviridae*** and ***Myoviridae*** families using Progressive MAUVE**. The names of the different bacteriophages are indicated under their maps. Colored blocks correspond to regions of nucleotide similarity which is indicated by its height and regions with a lack of homology are outside these blocks or indicated in white inside the blocks. The tRNA genes are indicated by the filled gene blocks.

Comparison of the genome of UAB_Phi20 with those of P22, ST64T, and ST104 at protein level using CoreGenes (Turner et al., 2013) revealed that shared 72% of its proteins with P22 and 63–65% with those of ST64T, and ST104. These results agree with that obtained by BlastP with protein-by-protein comparison (Table 1) and allow classifying UAB_Phi20 into *P22likevirus* genus as sharing at least 40% of proteins is a requisite to be classified into a determined genus (Lavigne et al., 2008). Finally, alignment of the annotated genomes of these bacteriophages using Mauve demonstrated the considerable sequence similarity between UAB_Phi20 and P22. Few noticeable differences with respect to ST64T and ST104 bacteriophages, especially at region 34–40 kb on the UAB_Phi20 genome, were observed (Figure 2). The high similarities between their genes, their organization and the identification of hypothetical genes lacking similarity with the above-mentioned bacteriophages demonstrate the genome mosaicism of these members of the *Podoviridae*. The origin of this genetic mosaicism agrees with the model of modular evolution of bacteriophages in which the horizontal transfer of genetic modules and their incorporation by homologous recombination leads to new genetic combinations that give rise to new lambdoid bacteriophages (Thomson et al., 2004).

### Genome analysis of UAB_Phi78

**Figure 3:**
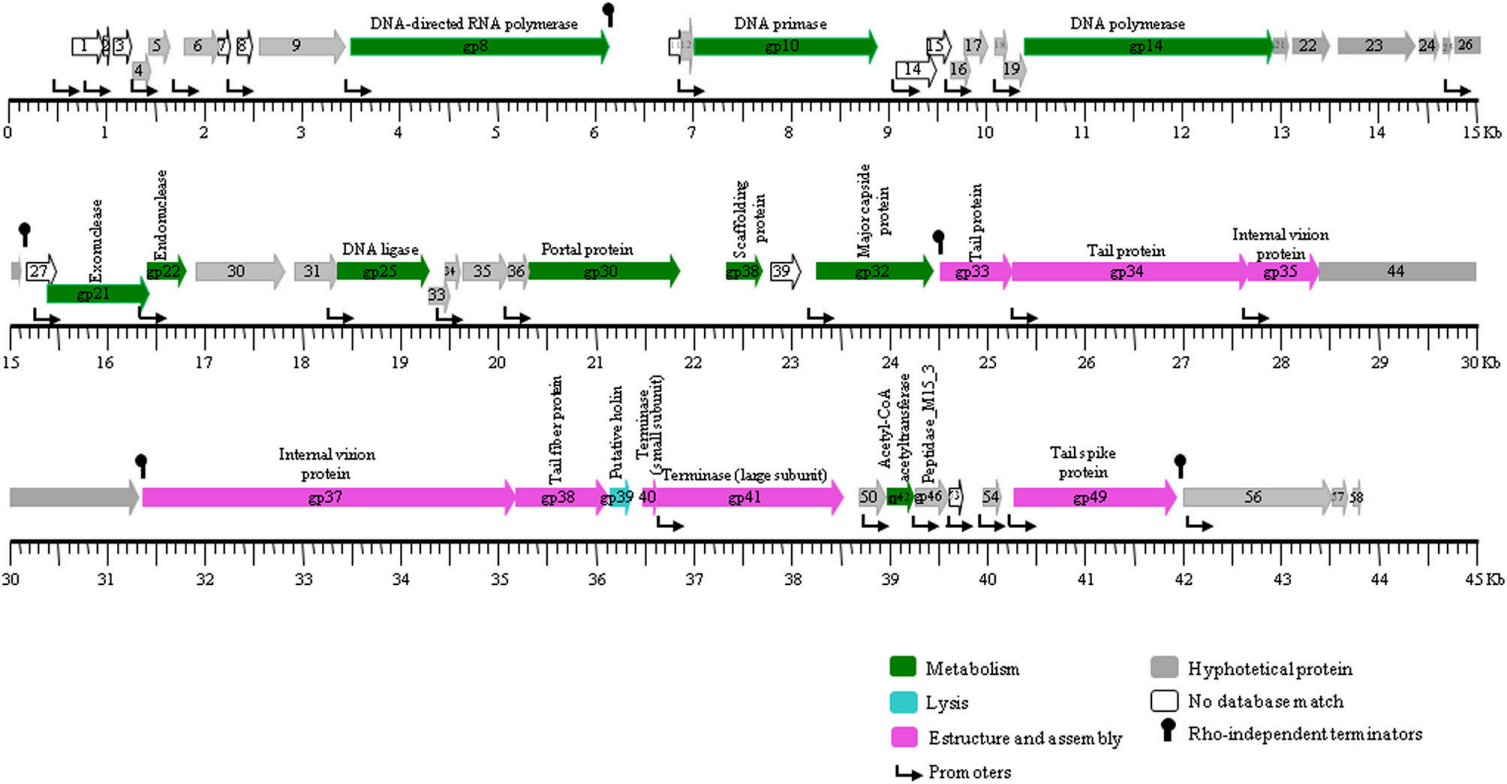
**Genomic structure of UAB_Phi78 including the Rho-indepedent terminators**. Arrows represent genes, and the different colors identify the functional category into which the homologous genes were classified. Gene functions are indicated where they are known. The color code for gene function is provided at the bottom of the figure. ORFs are numbered consecutively from left to right as described in Table 2, and are indicated by arrows pointing to the direction of transcription.

The UAB_Phi78 genome is a linear dsDNA molecule of 44,110 bp including DTR of 179 bp and with a G+C content of 47.41%, slightly lower than that of *Salmonella* (52.2%). Genome analysis predicted 58 putative ORFs (Table 2, Figure 3). The genome annotation of the SP6 bacteriophage (Genbank accession number NC_004831) was used to assign similarities to UAB_Phi78 ORFs because the genome of UAB_Phi78 showed the highest similarity (86%) with the genome of this phage after analysis with ClustalW2 program.

**Table 2:** **Features of bacteriophage UAB_Phi78 genome, ORFs, gene products, and functional assignments**.

**ORF**	**Gene**	**Position (nt)**		**No of amino acids**	**Predictive function**	**Closest hit (Accession number)**	**% Amino acid identity**	**Best *e*-value**
		**From**	**To**					
1		662	949	95	Unknown			
2		954	1016	20	Unknown			
3		1062	1241	59	Unknown			
4		1238	1417	59	Unknown	Gp3 Bacteriophage SP6 (AAP48742.1)	88	4.E-28
5		1410	1619	69	Unknown	Gp4 [Bacteriophage SP6] (AAP48743.1)	76	5.E-25
6		1773	2129	118	Unknown	Gp5 [Bacteriophage SP6] (AAP48744.1)	75	3.E-36
7		2130	2249	39	Unknown			
8		2319	2474	51	Unknown			
9		2537	3412	291	Unknown	Gp6 [Bacteriophage K1E] (CAJ29406.1)	72	2.E-142
10	*gp8*	3487	6111	874	DNA-directed RNA polymerase	Gp8 [Bacteriophage SP6] (AAP48747.1)	98	0
11		6771	6845	24	Unknown			
12		6849	6974	41	Unknown	gene 1.1 [Bacteriophage T7] (AAP33970.1)	65	0.012
13	*gp10*	6976	8871	631	DNA primase	Gp10 [Bacteriophage SP6] (AF159357.1)	98	0
14		9065	9469	134	Unknown			
15		9381	9614	77	Unknown			
16		9607	9807	66	Unknown	Gp11 [Bacteropphage SP6] (AAP48750.1)	89	2.E-34
17		9758	10000	80	Unknown	Gp12 [Bacteriophage SP6] (AAP48751.1)	93	9.E-27
18		10068	10172	33	Unknown	Gp11.5 [Bacteriophage K1E] (CAJ29415.1)	59	0.004
19		10159	10377	72	Unknown	Gp12 [Bacteriophage K1E] (CAJ29416.1)	93	3.E-42
20	*gp14*	10364	12910	848	DNA polymerase	Gp14 [Bacteriophage SP6] (AAP48753.1)	95	0
21		12910	13008	32	Unknown	Gp15 [Bacteriophage SP6] (AAP48754.1)	75	6.E-07
22		13123	13500	125	Unknown	Gp17 [Bacteriophage SP6] (AAP48756.1)	64	2.E-47
23		13580	14389	269	Unknown	Gp18 [Bacteriophage SP6] (AAP48757.1)	92	6.E-180
24		14407	14625	72	Unknown	Gp19 [Bacteriophage SP6] (AAP48758.1)	100	4.E-45
25		14645	14728	27	Unknown			
26		14731	15099	122	Unknown	Gp20 [Bacteriophage SP6] (AAP48759.1)	92	1.E-62
27		15165	15476	103	Unknown			
28	*gp21*	15383	16414	343	Exonuclease	Gp21 [Bacteriophage SP6] (AAP48760.1)	96	0
29	*gp22*	16399	16809	136	Endonuclease	Gp22 [Bacteriophage SP6] (AAP48761.1)	97	4.E-90
30		16895	17809	304	Unknown	Gp23 [Bacteriophage SP6] (AAP48762.2)	98	0
31		17910	18359	149	Unknown	23 [Bacteriophage K1-5] (AAR90065.1)	63	4.E-53
32	*gp25*	18359	19306	315	DNA ligase	Gp25 [Bacteriophage SP6] (AAP48764.1)	96	0
33		19278	19493	71	Unknown	Gp26 [Bacteriophage SP6] (AAP48765.1)	98	6.E-35
34		19456	19599	47	Unknown	Gp27 [Bacteriophage SP6] (AAP48766.1)	90	1.E-09
35		19629	20090	153	Unknown	Gp28 [Bacteriophage SP6] (AAP48767.1)	98	6.E-106
36		20100	20309	69	Unknown	Gp29 [Bacteriophage SP6] (AAP48768.1)	99	9.E-38
37	*gp30*	20311	21858	515	Portal protein	Gp30 [Bacteriophage SP6] (AAP48769.1)	99	0
38	*gp31*	22329	22706	125	Scaffolding protein	Gp31 [Bacteriophage SP6] (AAP48770.1)	92	2.E-71
39		22782	23078	98	Unknown			
40	*gp32*	23256	24458	400	Major capside protein	Gp32 [Bacteriophage SP6] (AAP48771.1)	98	0
41	*gp33*	24514	25254	246	Tail protein	Gp33 [Bacteriophage SP6] (AAP48772.1)	98	0
42	*gp34*	25254	27677	807	Tail protein	Gp34 [Bacteriophage SP6] (AAP48773.1)	98	0
43	*gp35*	27668	28387	239	Internal virion protein	Gp35 [Bacteriophage SP6] (AAP48774.1)	98	1.E-165
44		28388	31324	978	Unknown	Gp36 [Bacteriophage SP6] (AAP48775.1)	99	0
45	*gp37*	31391	35203	1270	Internal virion protein	Gp37 [Bacteriophage SP6] (AAP48776.1)	99	0
46	*gp38*	35203	36162	319	Tail fiber protein (adaptor)	Gp38 [Bacteriophage SP6] (AAP48777.1)	98	0
47	*gp39*	36171	36365	64	Putative holin	Gp39 [Bacteriophage SP6] (AAP48778.1)	97	2.E-35
48	*gp40*	36505	36651	48	Terminase (small subunit)	Gp40 [Bacteriophage SP6] (AAP48779.1)	96	4.E-23
49	*gp41*	36651	38549	632	Terminase (large subunit)	Gp41 [Bacteriophage SP6] (AAP48780.1)	97	0
50		38702	38977	91	Unknown	Gp41 [Bacteriophage K1E] (CAJ29452.1)	76	2.E-40
51	*gp42*	38993	39280	95	Putative Acetyl-CoA acetyltransferase	Gp42 [Bacteriophage K1E] (CAJ29453.1)	83	8.E-47
52	*gp46*	39283	39627	114	Peptidase_M15_3	Gp46 [Bacteriophage SP6] (AAP48785.1)	75	4.E-60
53		39627	39764	45	Unknown			
54		39981	40166	61	Unknown	Gp45 [Bacteriophage K1E] (CAJ29456.1)	88	6.E-30
55	*gp49*	40297	41949	550	Tail spike protein	Gp49 [Bacteriophage SP6] (AAP48788.1)	98	0
56		42033	43547	504	Unknown	Gp50 [Bacteriophage SP6] (AAP48789.1)	96	0
57		43555	43698	47	Unknown	Gp51 [Bacteriophage SP6] (AAP48790.1)	96	3.E-22
58		43752	43829	25	Unknown	Gp52 [Bacteriophage SP6] (AAP48791.2)	100	3.E-19

Gene numbers correspond with their predicted function, if known, followed by the nature of the evidence that supports the functional classification. Genes with no functional prediction, but with significant sequence similarity to genes in the NCBI database as determined by BLASTP are also listed.

A BPROM search identified 25 promoters (Table S1). Each had a −10 and −35 consensus sequences, ggTAtaaT and TTGAca, respectively (the conserved bases are indicated in capital letters). Four potential Rho-factor independent terminators were also identified in the UAB_Phi78 genome with FindTerm program (Figure 3). The first was located after gene encoding the RNA polymerase (*gp8*); the second and fourth immediately after genes encoding a protein of unknown function, and the third downstream of the gene (*gp32*) encoding the major capsid protein. Additionally, a fifth terminator was identified with the Transterm program. This was located after the genes encoding the tail spike protein. For 57 of the 58 predicted genes ATG was the translation initiation codon; in the remaining gene, *orf39*, the start codon was TTG. TAA was the most prevalent (67.2%) stop codon, followed by TGA and TAG (19 and 13.8%, respectively).

Among the 58 ORFs, 20 could be assigned functions and showed significant similarity with reported proteins of the SP6 bacteriophage (Dobbins et al., 2004). Hypothetical proteins were encoded by 26 ORFs whereas 12 did not show similarity with any gene product of the databases. According to a homology-search-based annotation the ORFs of UAB_Phi78 were categorized into three functions. Within the metabolic functions, the protein encoded by *orf20* showed significant identity (95%) with the DNA polymerase encoded by gene SP6 *gp14*, suggested to be the origin of bidirectional replication in SP6 (Dobbins et al., 2004). Likewise, proteins associated with the DNA metabolism of the phage genome were also identified: RNA polymerase (Gp8), DNA primase (Gp10), exonuclease (Gp21), endonuclease (Gp22), and DNA ligase (Gp25). All of them showed an identity of ~95% with their counterparts in the SP6 genome (Dobbins et al., 2004). It is remarkable that this phage encodes a RNA polymerase that may control the expression of its own DNA polymerase, similar to that described for the phage T7 (Kropinski et al., 2007). This could promote an efficient transcription of UAB_Phi78 genes and justify that the timing of the latent period was significantly lower than that of the other two phages studied in this work (Bardina, 2011; Spricigo, 2011). Accordingly, by PCR amplification, the UAB_Phi78 DNA was detected 10 min after infection of bacterial cells, whereas the UAB_Phi20 and UAB_Phi87 DNA were seen 20 min after infection (data not shown).

Finally, it is noteworthy that protein encoded by *orf6* of the UAB_Phi78 bacteriophage has 75% identity with SP6 Gp5 protein which encodes a putative anti-restriction protein. It has been suggested that it is the responsible for phage multiplication in *Salmonella* cells with or without its natural type I restriction systems (Scholl et al., 2004). Obviously, this can confer an advantage over other bacteriophages.

With respect to lysis, the *orf47* encoded a protein with a 96% identity with a putative holin codified in the SP6 *gp39* gene. Similar to the results in SP6, K1E, and K1–5 bacteriophages, no endolysin homologous to that encoding T7*gp18.5* and involved in lysis was identified (Dobbins et al., 2004; Scholl et al., 2004). This gene has only been identified in the genome of bacteriophage K1F (Scholl and Merril, 2005) and its expression is necessary only for cell lysis in the presence of high concentrations of divalent cations (Dobbins et al., 2004). It is worth mentioning that protein encoded by *orf44* had a 99% identity with SP6 Gp36 protein, whose C-terminal sequence showed a slight similarity with that of cell wall lysozymes and its lysozyme activity differed from that of the typical endolysins of similar bacteriophages (Dobbins et al., 2004; Scholl et al., 2004). Moreover, ORF52 had a 75% identity with SP6 Gp46 protein, recently identified as a peptidase_M15_3 (Oliveira et al., 2013). This protein, also identified in both K1E and K1-5 phages, is suggested to be an endolysin although without biochemical evidence.

Proteins involved in structure and assembly were encoded in more than half of the UAB_Phi78 genome, from approximately *orf37* to *orf55* (Table 2; Figure 3). Terminases (Gp40 and Gp41), head portal (Gp30), internal virion (Gp35 and Gp37), tail (Gp33 and Gp34), tail fiber (Gp38), and tail spike (Gp49) proteins were detected in this region, showing a ≥91% similarity with the corresponding proteins of the SP6 bacteriophage. However, three proteins encoded in this region (ORF50, ORF51, and ORF54) showed the highest identity (>75%) with hypothetical proteins of the K1E bacteriophage (Scholl et al., 2004) but, no homology was found for protein encoded by *orf53* in any database.

UAB_Phi78 has the protein Gp49 and the hypothetical protein encoded by *orf56*, with a high identity to the counterparts proteins of SP6 (Gp 49 and Gp50 proteins; Table 2) which have been predicted as receptor-binding proteins able to interact with two distinct receptors in the polysaccharide. SP6 Gp49 protein must interact with the *Salmonella* O-antigen because is closely related to the P22 tail spike protein (Gp9) with endorhamnosidase activity that cleaves the α 1,3-O-glycosidic bond between the repeating tetrasaccharide units of this antigen (Iwashita and Kanegasaki, 1976; Scholl et al., 2004). The second receptor, distinct from O-antigen and recognized by Gp50, was predicted for SP6 bacteriophage because this phage infected a *galE* mutant of *S*. Typhimurium LT2 (Scholl et al., 2004; Nguyen et al., 2012). Similarly, we hypothesized that bacteriophage UAB_Phi78 would recognize two receptors. In this sense, the bacteriophage UAB_Phi78 infected *galE* mutant of *S*. Typhimurium LT2 but not deep rough (*rfa*) mutants (data not shown). It must be noted that the two other bacteriophages studied here did not infect those mutants (data not shown).

After analysis using CoreGenes (Turner et al., 2013), UAB_Phi78 and SP6 bacteriophages have ~83% of proteins in common. The Rho-independent terminators were in the same position in both genomes, although their sequences showed <56% similarity (Dobbins et al., 2004). The main differences between the two bacteriophages occur at the beginning of the sequence of the UAB_Phi78 genome and in the region between DNA primase and DNA polymerase, where there are many genes encoding proteins without defined functions according to the NCBI databases, including a hypothetical protein with unknown function that is also present in bacteriophage K1E (Gp12). Moreover, UAB_Phi78 shared 80 and 69%, respectively of its proteome with those of *E. coli* bacteriophages as K1-5 and K1E.

Therefore, UAB_Phi78 belongs to *Sp6likevirus* genus of the *Podoviridae* family (Lavigne et al., 2008), which includes >35% of *Salmonella* bacteriophages (Abedon et al., 2011). An alignment of the annotated genomes of these four bacteriophages using Mauve reveals that their shared genes are largely collinear, with few noticeable differences at ~1–2, 6.7, 22, and 39 Kb on the UAB_Phi78 genome with respect to the others (Figure 2).

### Genome analysis of UAB_Phi87

**Figure 4:**
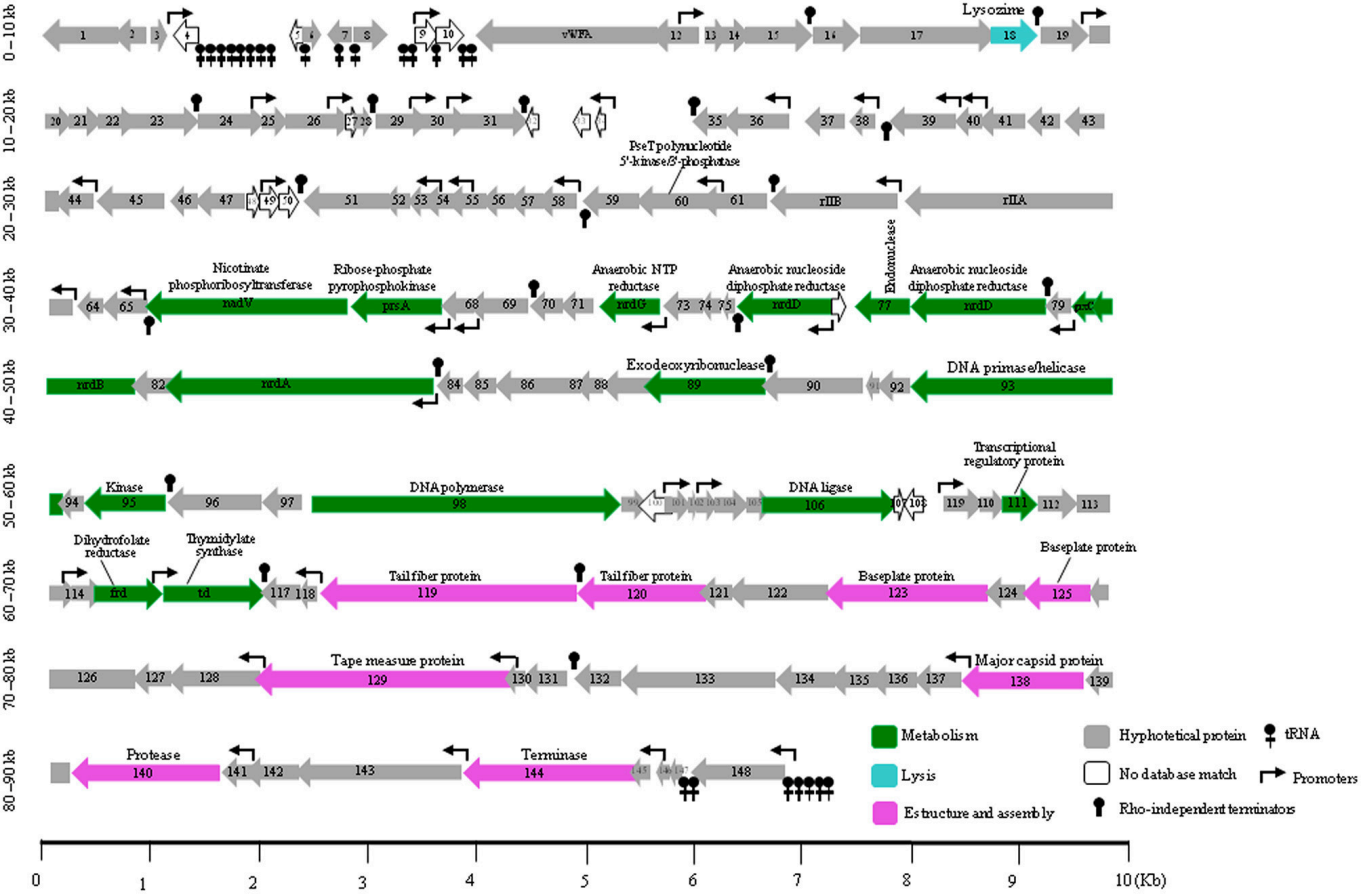
**Genomic structure of UAB_Phi87, including the Rho-independent terminators and tRNAs**. Arrows represent genes, and the different colors identify the functional category into which the homologous genes were classified. Gene functions are indicated where they are known. The color code for gene function is provided at the bottom of the figure. ORFs are numbered consecutively from left to right as described in Table 3, and are indicated by arrows pointing to the direction of transcription.

The complete sequenced genome of UAB_Phi87 consisted of 87,669 bp, with DTR of 608 bp and with a G+C percentage of 38.9%, clearly lower than that of *Salmonella* (52.2%). The UAB_Phi87 genome contained 210 putative ORFs, of which 148 were finally selected (Table 3); the remaining 62 were in regions that overlap with these 148 ORFs. Putative functions could be assigned only to 29 (19%) of the 148 ORFs based on protein sequence similarities. The other 119 ORFs consisted of hypothetical proteins without assigned function. Of these, 104 showed high similarities with hypothetical proteins of bacteriophages Felix O1 of *Salmonella*, wV8 of *E. coli*, and in a lesser extent of *Salmonella* FSL SP107, FSL SP010, and FSL SP012. Fifteen of these 119 ORFs were apparently unique to UAB_Phi87 and they lacked similarity with sequences deposited in the databases. Potential Shine-Dalgarno sequences were highly conserved (AGGAGGA) and, the mean distance between this consensus sequence and the majority of RBS was 14 bp. Up to 42 hypothetical promoters, with highly conserved consensus sequences at −10 (TATAAT) and −35 (TTGACA), were detected (Table S1). The high degree of conservation of these sequences and their similarity with those of prokaryote promoters could be a general advantage for phage, as following infection they would be recognized by host bacteria. Twenty Rho-independent terminators were identified by FindTerm (Figure 4). Almost all of the ORFs (146 out of 148) started with an ATG codon; the exceptions were *orf118* and *orf144*, in which TTG was the start signal. As for the stop codons, most ORFs contained a TAA codon (67.1%). TGA was present in 25.5% of the remaining ORFs and TAG in 7.4%. The genome of UAB_Phi87 contains 23 tRNA genes and three of them (13, 18, and 20) may code pseudo-tRNA (Table S2). From the 20 functional tRNAs, 9 were found to be present at a frequency 1.5 times higher in the phage than in *Salmonella*. The high number of tRNA has been also documented in other *Felixounalikeviruses* (Whichard et al., 2010). Their presence seems to compensate for differences in codon usage between the phage and the host and to enable a positive impact on translation of phage-derived mRNA and its infectivity (Bailly-Bechet et al., 2007). Moreover, and similar to bacteriophage Felix O1, the presence of a Met tRNA suggested a positive role for this tRNA in translational initiation in phage-infected cells (Whichard et al., 2010).

**Table 3:** **Features of bacteriophage UAB_Phi87 genome, ORFs, gene products, and functional assignments**.

**ORF**	**Gene**	**Position (nt)**		**Strand**	**No of amino acids**	**Predictive function**	**Closest hit (Accession number)**	**% Amino acid identity**	**Best *e*-value**
		**From**	**To**						
1		767	210	−	185		Hypothetical protein wV8_gp055 (YP_002922836.1)	97	7.E-129
2		1006	764	−	80		Hypothetical protein [*Escherichia* phage EC6] (YP_009151266.1)	88	1.E-44
3		927	1037	+	36		Hypothetical protein wV8_gp054 (YP_002922835.1)	70	7.E-04
4		1432	1250	−	60				
5		2479	2384	−	31				
6		2460	2600	+	46		Hypothetical protein wV8_gp050 (YP_002922831.1)	100	2.E-24
7		2978	2799	−	59		Hypothetical protein wV8_gp049 (YP_002922830.1)	100	6.E-36
8		3045	3302	+	85		Hypothetical protein wV8_gp048 (YP_002922829.1)	96	2.E-50
9		3693	3869	+	58				
10		3820	4035	+	71				
11	*vWFA*	5695	4280	+	471		Hypothetical protein wV8_gp047 (YP_002922828.1)	98	0
12		6147	5776	−	123		Hypothetical protein wV8_gp046 (YP_002922827.1)	98	8.E-80
13		6397	6558	+	53		Hypothetical protein Felix01p077 (YP_001504372.1)	96	5.E-29
14		6540	6773	+	77		Hypothetical protein Felix01p076 (NP_944854.1)	96	4.E-47
15		6767	7357	+	196		Hypothetical protein wV8_gp043 (YP_002922824.1)	95	3.E-123
16		7405	7776	+	123		Hypothetical protein wV8_gp042 (YP_002922823.1)	86	2.E-73
17		7773	8906	+	377		Hypothetical protein wV8_gp041 (YP_002922822.1)	93	0
18		8906	9370	+	154	Lysozyme	Lysin (lysozyme) [*Salmonella* phage FelixO1] (NP_944846.1)	99	2.E-108
19		9421	9819	+	132		Hypothetical protein Felix01p068 (NP_944844.1)	97	4.E-89
20		9812	10210	+	132		Hypothetical protein SP107_00535 [*Salmonella* phage FSL SP-107](AGF89476)	94	6.E-85
21		10210	10503	+	97		Hypothetical protein wV8_gp037 (YP_002922818.1)	97	7.E-62
22		10496	10840	+	114		Hypothetical protein wV8_gp036 (YP_002922817.1)	100	5.E-76
23		10840	11421	+	193		Hypothetical protein [*Salmonella* phage SBA-1781] (AFU63462.1)	98	4.E-138
24		11494	12039	+	181		Hypothetical protein SP107_00555 [*Salmonella* phage FSL SP-107] (YP_009219564.1)	86	5.E-112
25		12036	12254	+	72		Hypothetical protein Felix01p056 (NP_944832.1)	92	2.E-41
26		12251	12754	+	167		Hypothetical protein SP010_00552 [*Salmonella* phage FSL SP-010] (AGF88761.1)	96	7.E-116
27		12736	12831	+	31				
28		12831	13055	+	74		Hypothetical protein wV8_gp031 (YP_002922812.1)	99	7.E-42
29		13110	13538	+	142		Hypothetical protein wV8_gp030 (YP_002922811.1)	56	3.E-46
30		13528	13992	+	154		Hypothetical protein Felix01p051 (NP_944827.1)	91	4.E-98
31		13949	14554	+	201		Hypothetical protein Felix01p050 (NP_944826.2)	91	3.E-106
32		14632	14525	−	35				
33		15166	15017	−	49				
34		15312	15235	−	25				
35		16494	16246	−	82		Hypothetical protein Felix01p049 (NP_944825.1)	100	9.E-53
36		17090	16560	−	176		Hypothetical protein SP010_00705 [*Salmonella* phage FSL SP-010] (AGF88787.1)	91	1.E-113
37		17653	17312	−	113		Hypothetical protein Felix01p044(NP_944820.1)	88	4.E-69
38		17979	17746	−	77		Hypothetical protein wV8_gp022 (YP_002922803.1)	92	4.E-45
39		18588	18046	−	180		Hypothetical protein HB2014_24 [*Salmonella* phage HB-2014] (YP_009146269.1)	96	3.E-123
40		18877	18674	−	67		Hypothetical protein wV8_gp020 (YP_002922801.1)	95	5.E-35
41		19386	18982	+	134		Hypothetical protein wV8_gp019 (YP_002922800.1)	91	6.E-84
42		19746	19474	−	90		Hypothetical protein wV8_gp018 (YP_002922799.1)	92	6.E-55
43		20169	19837	−	110		Hypothetical protein [*Salmonella* phage SBA-1781] (AFU63421.1)	95	2.E-65
44		20459	20163	−	98		Hypothetical protein Felix01p034 (NP_944810.1)	98	5.E-63
45		21064	20552	−	170		Hypothetical protein SP010_00685 [*Salmonella* phage FSL SP-010](AGF88783.1)	96	2.E-116
46		21412	21155	−	85		Hypothetical protein wV8_gp015 (YP_002922796.1)	70	4.E-31
47		21882	21499	−	127		Hypothetical protein wV8_gp014 (YP_002922795.1)	93	9.E-82
48		21794	21901	+	35				
49		22010	22168	+	52		Hypothetical protein SP012_00635 [*Salmonella* phage FSL SP-012] (AGF88904.1)	98	3.E-27
50		22170	22325	+	51				
51		23189	22404	−	261		Phage conserved protein Felix01p025 (NP_944801.1)	98	0
52		23390	23190	−	66		Hypothetical protein wV8_gp011 (YP_002922792.1|)	98	6.E-40
53		23610	23383	−	75		Hypothetical protein Felix01p021 (NP_944797.1)	85	2.E-38
54		23941	23585	−	118		Hypothetical protein Felix01p019 (NP_944795.1)	96	3.E-60
55		24215	23901	−	104		Hypothetical protein Felix01p017 (NP_944793.2)	100	6.E-69
56		24481	24212	−	89		Hypothetical protein wV8_gp008 (YP_002922789.1)	99	5.E-59
57		24747	24478	−	89		Hypothetical protein Felix01p015 (NP_944791.1)	99	2.E-56
58		24988	24641	−	115		Phage conserved protein Felix01p014 (NP_944790.1)	100	3.E-78
59		25505	25041	−	154		Hypothetical protein wV8_gp005 (YP_002922786.1)	100	2.E-104
60		26211	25516	−	231	PseT polynucleotide 5′-kinase/3′-phosphatase	Putative PseT polynucleotide 5′-kinase/3′-phosphatase [*Salmonella* phage FSL SP-010] (AGF88668.1)	99	2.E-166
61		26737	26189	−	182		Hypothetical protein wV8_gp003 (YP_002922784.1)	99	2.E-128
62	*rIIB*	27947	26838	−	369		rIIB protein [*Escherichia* phage wV8] (YP_002922783.1)	98	0
63	*rIIA*	30393	28027	−	788		rIIA protein [*Salmonella* phage FSL SP-010] (AGF88671.1)	98	0
64		30598	30422	−	58		Hypothetical membrane protein Felix01p243 (NP_945023.1)	95	8.E-32
65		30915	30580	−	111		Hypothetical protein SP010_00075 [*Salmonella* phage FSL SP-010] (AGF88673.1)	97	4.E-73
66	*nadV*	32750	30969	−	593	Nicotinate phosphoribosyltransferase	Putative nicotinate phosphoribosyltransferase [*Salmonella* phage FSL SP-107](AGF89421.1)	96	0
67	*prsA*	33677	32796	−	293	Ribose-phosphate pyrophosphokinase	Putative ribose-phosphate pyrophosphokinase [*Salmonella* phage FSL SP-107] (AGF89420.1)	99	0
68		33971	33693	−	92		Hypothetical protein Felix01p233 (NP_945013.1)	96	1.E-59
69		34479	33964	−	171		Hypothetical protein SP10700240 [*Salmonella* phage FSL SP-107] (AGF89418.1)	96	8.E-121
70		34851	34531	−	106		Hypothetical protein Felix01p227 (NP_945007.1)	97	8.E-67
71		35111	34854	−	86		Hypothetical protein wV8_gp132 (YP_002922914.1)	99	6.E-52
72	*nrdG*	35721	35236	−	161	Anaerobic NTP reductase	NrdG, small subunit [*Escherichia* phage wV8] (YP_002922912.1)	95	2.E-112
73		36176	35781	−	131		Hypothetical protein SP10700210 [*Salmonella* phage FSL SP-107] (AGF89412.1)	95	5.E-89
74		36373	36173	−	66		Hypothetical membrane protein Felix01p221 (NP_945001.1)	97	2.E-37
75		36474	36349	−	41		Hypothetical membrane protein Felix01p220 (NP_945000.2)	90	3.E-17
76	*nrdD*	37382	36528	−	284	Anaerobic nucleoside diphosphate reductase	NrdD [*Escherichia* phage wV8] (YP_002922907.1)	99	0
77		38102	37638	−	154	Homing endonuclease	Homing endonuclease [*Salmonella* phage HB-2014] (YP_009146359.1)	96	2.E-105
78	*nrdD*	39414	38215	−	399	Anaerobic nucleoside diphosphate reductase	NrdD [*Escherichia* phage wV8] (YP_002922907.1)	99	0
79		39669	39463	−	68		Hypothetical membrane protein FelixO1p218 (NP_944998.1)	97	3.E-37
80	*grxC*	39904	39662	−	80	Glutaredoxin	Putative phage glutaredoxin [Phage FelixO1] (NP_944996.1)	95	2.E-50
81	*nrdB*	40977	39904	−	357	Ribonucleoside triphosphate reductase,	Ribonucleoside triphosphate reductase, beta chain [Phage FelixO1] (NP_944994.1)	100	0
82		41315	40974	−	113		Hypothetical protein WV8_gp121 [*Escherichia* phage wV8] (YP_002922903.1)	88	1.E-67
83	*nrdA*	43521	41287	−	744	Ribonucleoside triphosphate reductase,	Ribonucleoside triphosphate reductase, alpha chain [Phage FelixO1] (NP_944991.1)	99	0
84		43807	43568	−	79		Hypothetical protein Felix01p210 (NP_944989.1)	97	4.E-51
85		44222	43899	−	107		Hypothetical membrane protein Felix01p208 (NP_944987.2)	100	2.E-72
86		44958	44203	−	251		Hypothetical protein wV8_gp117 [*Escherichia* phage wV8] (YP_002922899.1)	98	0
87		45181	44951	−	76		Hypothetical protein wV8_gp116 [*Escherichia* phage wV8] (YP_002922898.1)	96	1.E-39
88		45700	45203	−	165		Hypothetical protein wV8_gp115 [*Escherichia* phage wV8] (YP_002922897.1)	100	2.E-117
89		46730	45690	−	346	Exodeoxyribonuclease	Putative exodeoxyribonuclease [*Salmonella* phage FSL SP-107] (AGF89399.1)	98	0
90		47650	46793	−	285		Hypothetical protein wV8_gp117 [*Escherichia* phage wV8] (YP_002922894.1)	99	0
91		47872	47723	−	49		Hypothetical protein Felix01p245 (YP_001504375.1)	100	1.E-25
92		48156	47869	−	95		Hypothetical protein Felix01p246 (YP_001504374.1)	93	5.E-57
93		50110	48125	−	661	DNA primase/helicase	Putative phage DNA primase/helicase [*Escherichia* phage wV8] (YP_002922891.1)	99	0
94		50303	50103	−	66		Hypothetical protein Felix01p187 (NP_944966.1)	100	2.E-36
95		51055	50312	+	247	Kinase	Putative deoxynucleotide monophosphate kinase [*Escherichia* phage HY02] (YP_009205000.1)	98	2.E-177
96		51918	51118	−	266		Hypothetical protein Felix01p181 (NP_944960.1)	97	0
97		52342	51920	−	140		Hypothetical protein Felix01p180 (NP_944959.1)	99	7.E-97
98		52569	55298	−	910	DNA polymerase	Putative DNA polymerase [*Escherichia* phage wV8] (YP_002922886.1)	99	0
99		55360	55578	+	72		Hypothetical protein JH2_060 [*Escherichia* phage JH2] (YP_009219503.1)	89	1.E-36
100		55813	55499	−	104				
101		55781	55999	+	72		Hypothetical protein SP010_00270 [*Salmonella* phage FSL SP-010] (AGF88712.1)	97	3.E-42
102		55996	56142	+	48		Hypothetical protein Felix01p244 (YP_001504373.1)	100	3.E-26
103		56165	56389	+	74		Hypothetical protein Felix01p170 (NP_944949.1)	89	7.E-43
104		56343	56606	+	87		Hypothetical protein Felix01p168 (NP_944947.1)	97	2.E-39
105		56603	56821	+	72		Hypothetical protein Felix01p166 (NP_944945.1)	99	4.E-45
106		56760	57896	+	378	DNA ligase	Putative DNA ligase [Phage FelixO1] (NP_944942.1)	98	0
107		57900	58046	+	48				
108		58114	57947	−	55				
119		58457	58840	+	127		Hypothetical protein Felix01p155 (NP_944938.1)	98	1-e-85
110		58842	59054	+	70		Hypothetical protein wV8_gp093 [*Escherichia* phage wV8] (YP_002922875.1)	97	8.E-41
111		59047	59346	+	99	Transcriptional regulatory protein	Putative transcriptional regulatory protein wV8_gp092 (YP_002922874.1)	98	1.E-65
112		59348	59707	+	119		Hypothetical protein wV8_gp091 (YP_002922873.1)	99	4.E-80
113		59721	60236	+	171		Hypothetical protein wV8_gp090 (YP_002922872.1)	98	9.E-41
114		60237	60497	+	86		Hypothetical protein wV8_gp089 (YP_002922871.1)	92	3.E-52
115	*frd*	60494	61039	+	181	Dihydrofolate reductase	Dihydrofolate reductase [*Escherichia* phage wV8] (YP_002922870.1)	94	8e–122
116	*td*	61041	61940	+	299	Thymidylate synthase	Thymidylate synthase [*Salmonella* phage FSL SP-107] (AGF89371.1)	99	0
117		62347	61976	-	123		Hypothetical protein wV8_gp086 (YP_002922868.1)	98	4.E-80
118		62541	62344	−	65		Hypothetical protein wV8_gp085 (YP_002922867.1)	100	9.E-36
119		64968	62620	−	782	Tail fiber protein	Putative tail fiber protein [Phage FelixO1] NP_944923.1)	77	0
120		66185	65019	−	388	Tail fiber protein	Putative tail fiber protein GP37 [Phage FelixO1] (NP_944921.1)	96	0
121		66490	66188	−	100		Hypothetical protein Felix01p141 (NP_944920.1)	99	2.E-65
122		67347	66490	−	285		Hypothetical protein Felix01p139 (NP_944918.1)	99	0
123		68819	67350	−	489	Baseplate protein	Putative baseplate component [*Salmonella* phage FSL SP-107] (AGF89443.1)	98	0
124		69238	68819	−	139		Phage conserved protein [Phage FelixO1] (NP_944914.1)	100	8.E-97
125		69861	69238	−	207	Baseplate protein	Putative baseplate protein [Phage FelixO1] (NP_944912.1)	99	1.E-152
126		70838	69861	−	325		Hypothetical protein wV8_gp077 (YP_002922859.1)	99	0
127		71179	70838	−	113		Hypothetical protein wV8_gp076 (YP_002922858.1)	100	5.E-77
128		71979	71179	−	266		Hypothetical protein wV8_gp075 (YP_002922857.1)	97	0
129		74210	71979	−	743	Tape measure domain	Hypothetical protein wV8_gp074 (YP_002922856.1)	99	0
130		74449	74210	−	79		Hypothetical protein Felix01p121 (NP_944900.1)	100	2.E-41
131		74850	74452	−	132		Hypothetical protein Felix01p120 (NP_944899.1)	100	6.E-89
132		75370	74924	−	148		Hypothetical protein wV8_gp071 (YP_002922853.1)	100	5.E104
133		76738	75386	−	450		Phage conserved structural protein [Phage FelixO1] (NP_944896.1)	97	0
134		77338	76739	−	199		Hypothetical protein Felix01p116 (NP_944895.1)	100	3.E-142
135		77714	77313	−	133		Hypothetical protein wV8_gp068 (YP_002922850.1)	99	1.E-92
136		78193	77711	−	160		Phage conserved protein [Salmonella phage FelixO1] (NP_944893.1)	99	5.E-111
137		78642	78193	−	149		Hypothetical protein Felix01p113 (NP_944892.1)	100	2.E-104
138		79767	78664	−	367	Major capsid protein	Major capsid protein [Phage FelixO1] (NP_944891.1)	99	0
139		80178	79801	−	125		Hypothetical protein Felix01p111 (NP_944890.1)	94	1.E-79
140		81536	80190	−	448	Protease	Putative head maturation protease [Phage FelixO1] (NP_944888.1)	99	0
141		81880	81548	−	110		Hypothetical protein Felix01p108 (NP_944887.1)	99	9.E-72
142		82380	81880	−	166		Hypothetical protein HB2014_56 [Salmonella phage HB-2014] (YP_009146299.1)	99	4.E-115
143		83846	82380	−	488		Hypothetical protein wV8_gp059 (YP_002922841.1)	99	0
144		85464	83863	−	533	Terminase	Terminase, large subunit [Phage FelixO1] (NP_944884.1)	100	0
145		85686	85486	−	66		Hypothetical protein wV8_gp057 (YP_002922839.1)	100	4.E-37
146		85887	85711	−	58				
147		85971	85879	−	31		Hypothetical protein Felix01p101 (NP_944880.1)	96	4.E-18
148		86819	86085	−	244		Hypothetical protein Felix01p100 (NP_944879.1)	97	4.E-166

Gene numbers correspond with their predicted function, if known, followed by the nature of the evidence that supports the functional classification.

Genes with no functional prediction, but with significant sequence similarity to genes in the NCBI database as determined by BLASTP are also listed.

The UAB_Phi87 ORFs encoding proteins with known functions were classified into three functional groups (Figure 4). The first one included proteins involved in nucleotide metabolism, which would allow phage replication and transcriptional control of the host machinery. Thus, DNA polymerase (*orf98*), DNA primase/helicase (*orf93*), DNA ligase (*orf106*), and other proteins encoded by genes *frd, nadV, nrdA, nrdB, nrdD, nrdG, prsA,* and *td* involved in the nucleotide metabolism were identified and presented an identity ≥94% with the counterpart proteins of the bacteriophages Felix O1 and FSL SP107 of *Salmonella*, and wV8 of *E. coli.* As it has been reported for some bacteriophages of *Felixounalikeviruses* genus (Moreno Switt et al., 2013), one split gene (*nrdD)* encoding the anaerobic ribonucleotide reductase was identified in UAB_Phi87. A gene (*orf77*) encoding a putative homing endonuclease interrupted the *nrdD* gene. In addition, this genetic structure also was in HB-2014 and JH2 phages of the *Myoviridae* family as we determined by bioinformatic analysis. The UAB_Phi87 homing endonuclease had an identity of 95–96% with the counterpart protein of all these bacteriophages and a 53% to that of the JSE bacteriophage, which belongs to the T4 group of bacteriophages infecting *E. coli*. Bacteriophages of this group, as T4 and JSE, and those of the *Felixounalikeviruses* genus typically possess several homing endonucleases (Whichard et al., 2010). Thus, T4 and Felix O1 bacteriophages encodes for 15 and 6 homing endonucleases, respectively. In contrast, in the UAB_Phi87 genome only a gene coding a homing endonuclease was identified.

The unique gene (*orf18*) with a clear function in lysis encoded a lysin with a 99% identity with the counterpart of Felix O1 bacteriophage. As in this phage, UAB_Phi87 lacks a holin gene adjacent to the lysin gene. Thus, as suggested for Felix O1 (Whichard et al., 2010), one as yet unidentified protein with unknown function located elsewhere in the UAB_Phi87 genome may assume that function. The UAB_Phi87 genome also contains *rIIA* and *rIIB* genes, first described in bacteriophage T4 (Miller et al., 2003), which encoded membrane-associated proteins of poorly understood function in this phage. It has been suggested that both could be indirectly involved in lysis inhibition, perhaps by perturbing membrane functions (Burch et al., 2011) when bacterial cells are reinfected by other T4 bacteriophages. It must be noted that UAB_Phi87 DNA was detected inside infected cells more than 100 min by PCR amplification (data not shown) which could be related to this phenomenon.

The third functional group contained structure and assembly proteins and included tail fiber (ORF119 and ORF120), baseplate (ORF123 and ORF125), tape measure (ORF129), major capsid (ORF138) proteins, and a putative head maturation protease (ORF140). All of them presented a high identity with their counterparts of FelixO1, FSL SP-107, and wV8 phages. Only, ORF119 showed lower identity (77%) with respect to the corresponding putative tail fiber of FelixO1 (Table 3). At difference of many phages and similar to Felix O1 phage, only a large terminase (ORF144) was identified in the UAB_Phi87 genome (Whichard et al., 2010) with a 100% of identity. As it had been reported these large terminases presented similarity with *Erwinia amylovora* ΦEa21-4 phage, and wV8 and rV5 phages which infected *E. coli* (Whichard et al., 2010).

After CoreGenes analysis, the proteome of UAB_Phi87 shared ≤90% with those of FelixO1 and wV8. This allows classifying UAB_Phi87 as belonging to *Felixounalikevirus* genus of *Myoviridae* family. A MAUVE comparison of these four genomes agrees with the results of protein-by-protein comparison, and revealed the mosaic structure of the UAB_Phi87 genome and also its high similarity in terms of both genetic content and functional organization with the genomes of the other bacteriophages (Figure 2).

### Determination of the genome ends of UAB_Phi20, UAB_phi78, and UAB_phi87 bacteriophages

Six types of ends are well-known in the lineal dsDNA contained in the tailed-bacteriophage virions: (i) single-stranded cohesive ends (*cos* ends), (ii) circularly permuted DTR, (iii) short, several hundred base pairs exact DTR, (iv) long, several thousand base pairs exact DTR, (v) terminal host sequences, and (vi) covalently bound terminal proteins (Casjens and Gilcrease, 2009). The first five types of ends are produced by the cleavage of DNA concatemers consequence of the phage DNA replication. These cleavages are closely tied with the phage DNA packaging due to terminases encoded by the phage itself.

After sequencing the genomes, we did not obtain a clear evidence of the ends of the chromosomes of UAB_Phi20, UAB_Phi78, and UAB_Phi87 bacteriophages. In order to clarify this and their packaging strategy, firstly, the DNA of the phages was obtained and digested with *Eco*RV enzyme. Afterwards the restriction product was heat treated prior electrophoresis. Results did not evidence any change of the restriction patterns of DNA treated and untreated with heat (data not shown), showing that the chromosome of these bacteriophages did not present *cos* ends.

**Figure 5:**
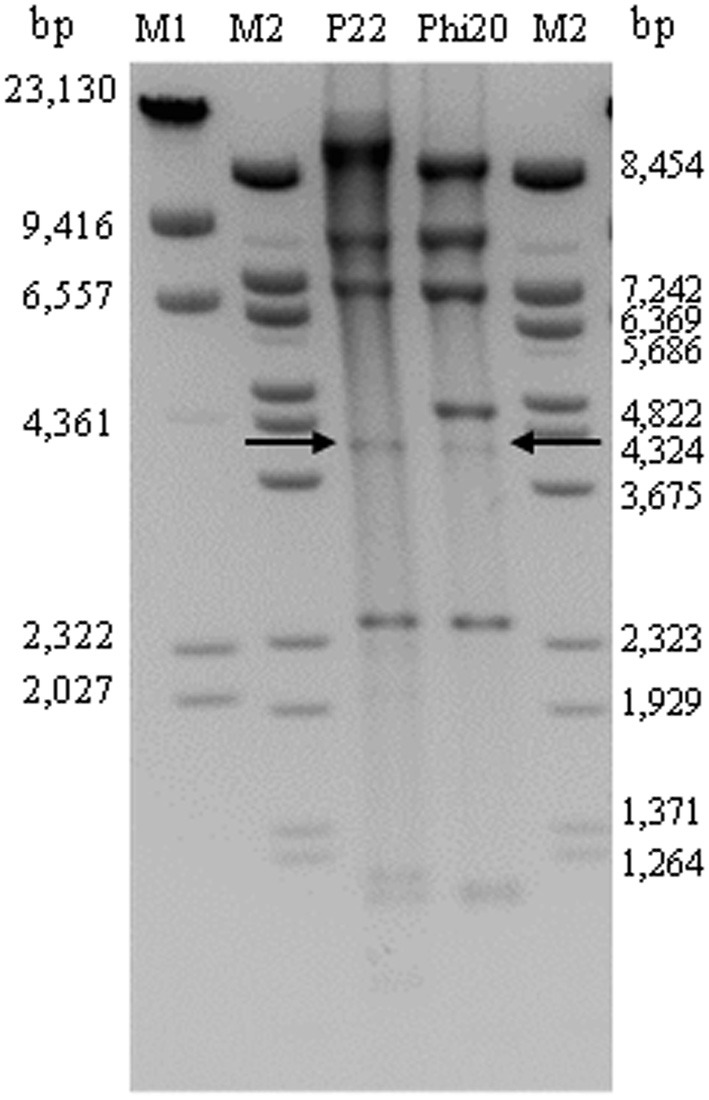
**Determination of genome ends of UAB_Phi20 phage after digestion with EcoRI enzyme**. Genome of bacteriophage P22 digested with *Eco*RI was used as control. Arrows indicate the 4007-bp fragment containing the *pac* sequence. Lambda DNA digested with *Hin*dIII (M1) or *Bst*EII (M2) were used as molecular markers. Sizes (bp) are indicated on both sides of the image.

**Figure 6:**
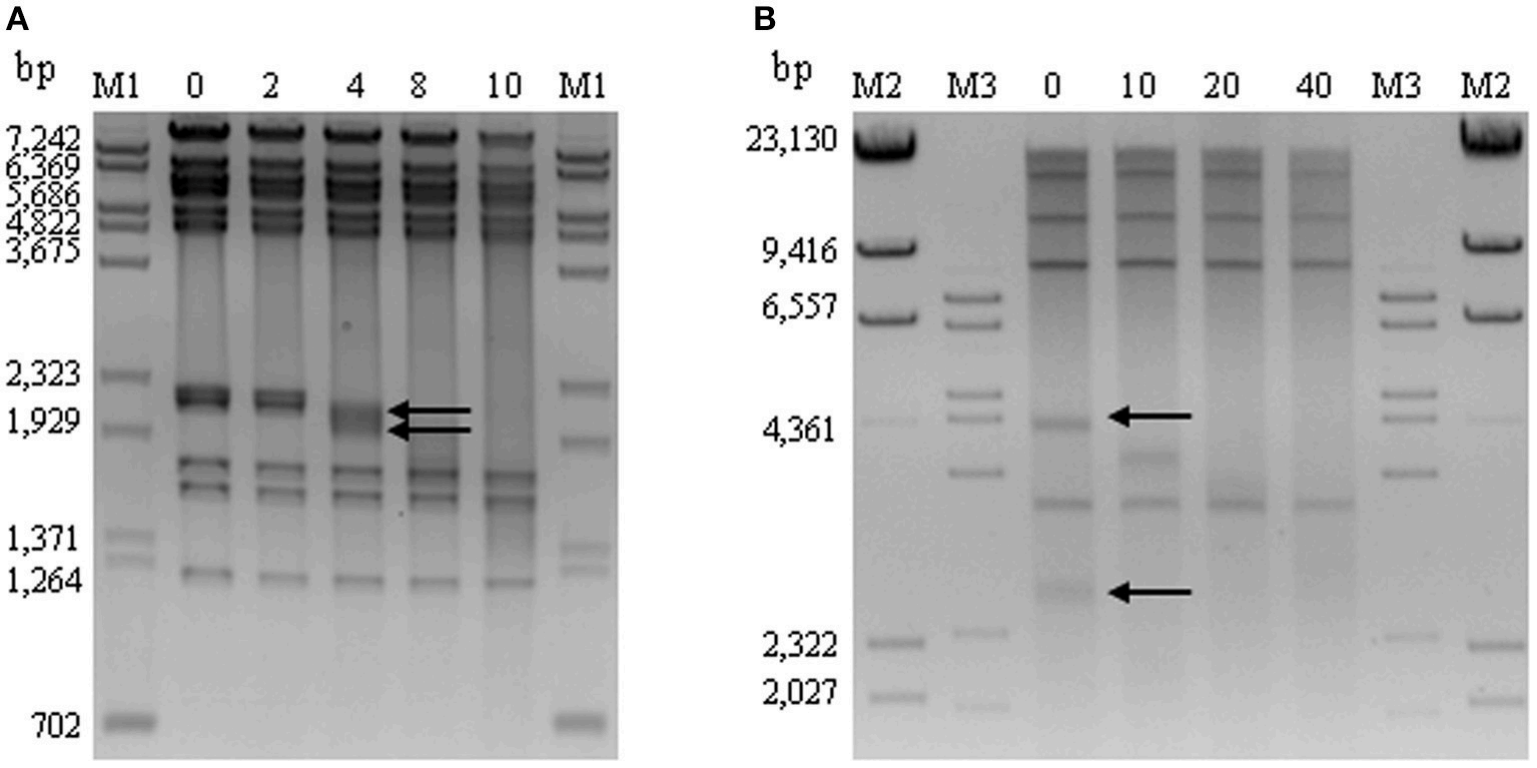
**Time-limited digestion with ***Bal***31 exonuclease of UAB_Phi78 and UAB_Phi87 DNA followed by digestion with ***Hin***dIII and ***Spe***I, respectively**. Arrows indicate the sequentially degraded DNA bands of 2200 and 2080 bp for UAB_Phi78 **(A)** and of 4322 and 2819 bp for UAB_Phi87 **(B)**. M: marker lanes containing a mixture of λ DNA digested with *Bst*EII and φX174 digested with *Hin*fI (M1), λ-DNA-digested *Hin*dIII (M2), and λ-DNA-digested *Bst*EII (M3). Sizes (bp) are indicated on the left side of the images.

Because of the high similarity of UAB_Phi20 genome with those of bacteriophages of the *P22likevirus* genus and the identification of a *pac* site in its genome, we believe that this phage would have circularly permuted DTR. This was confirmed by observing the under-representation of one 4007 bp DNA fragment (Figure 5), which would contain the *pac* sequence, in *Eco*RI digested genome. This result is expected for bacteriophages, as P22 phage, which presents this type of ends in their chromosomes (Casjens and Gilcrease, 2009) (Figure 5). Following, we studied if the chromosome ends of UAB_Phi78 and UAB_Phi87 bacteriophages presented DTR in the ends of their chromosomes. Time-limited treatment of their DNA with exonuclease *Bal*31 followed by digestion with *Hin*dIII and *Spe*I enzymes, respectively, revealed the disappearance of two fragments in their respective restriction patterns. Thus, in UAB_Phi78, two fragments of 2212 and 2109 bp were simultaneously degraded whereas in UAB_Phi87 the disappearance of two fragments of 4322 and 2819 bp was observed (Figure 6). These data indicated that the degraded fragments contained the chromosomal ends of both UAB_Phi78 and UAB_Phi87 bacteriophages. According to this, specific primers were designed and used for sequencing the recovered and purified restriction fragments as templates. The primers that displayed drop-offs of the sequencing signal were selected and used to confirm the genome end sequences. For this, the respective phage genome was used as template and typical sudden drop-offs of the sequencing signal were observed (Figure S1). The analysis of the sequences obtained allowed us to identify short DTR of 179 and 608 bp for UAB_Phi78 and UAB_Phi87, respectively (Figure S1). The size of the UAB_Phi78 DTR was similar to that described in bacteriophage SP6 (Dobbins et al., 2004; Scholl et al., 2004). Likewise, DTR of FelixO1 (Whichard et al., 2010) and FO1a (Marti, 2013) bacteriophages had a similar size to those of UAB_Phi87.

**Figure 7:**
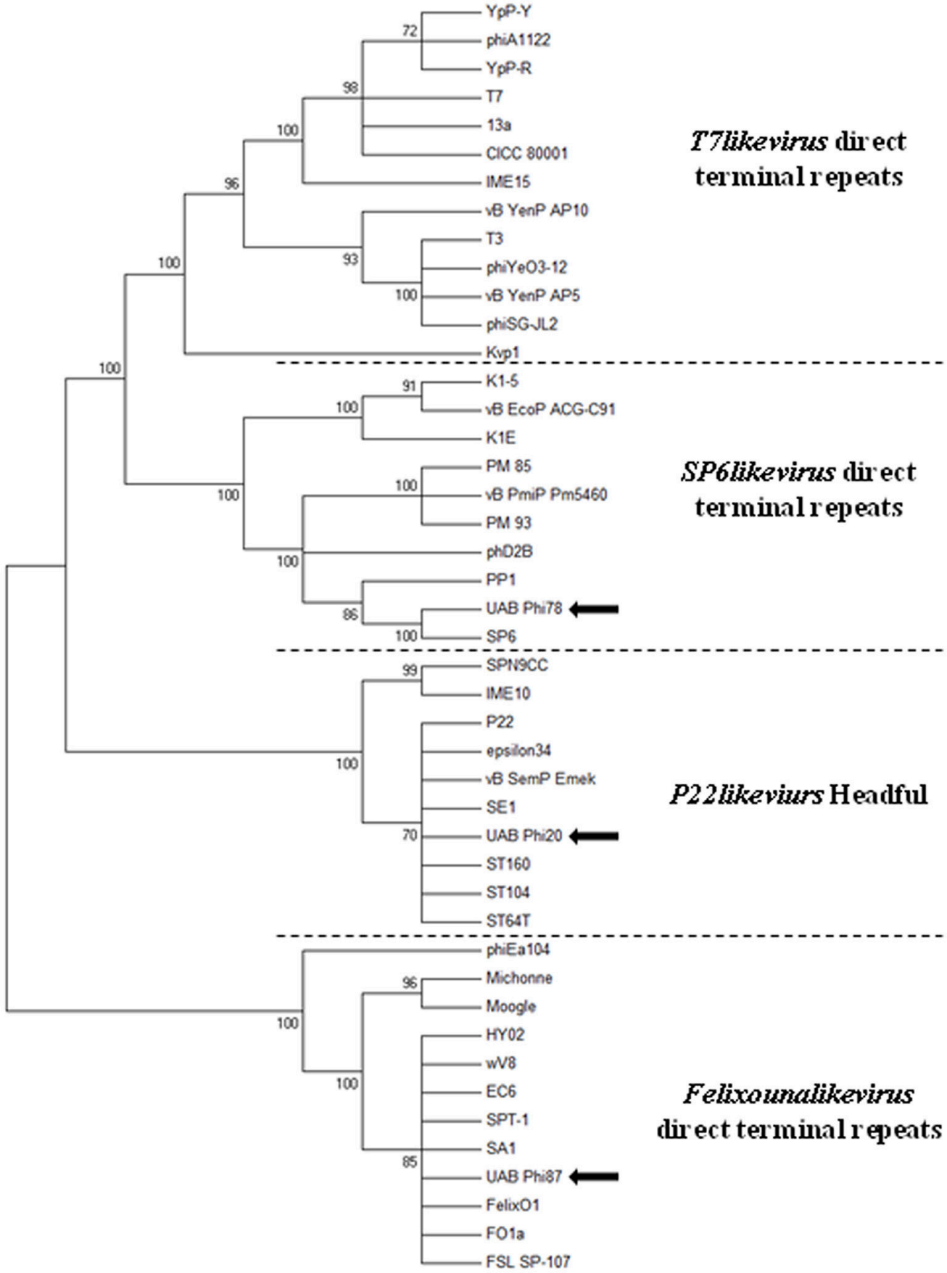
**Neighbor-joining phylogenetic tree of large terminase subunit sequences of bacteriophages UAB_Phi20, UAB_Phi78, and UAB_Phi87 (indicated by arrows) and comparison to other phages with known packaging mechanisms**. Bootstrap analysis was performed with 1000 repetitions. The node of phylogenetic tree shows the bootstrap confidence values above 70%.

It has been reported that the packaging mechanisms, and in consequence, the type of chromosome ends of bacteriophages can be predicted comparing the amino acid sequences of the known large terminase subunits with similar enzymatic end-generating functions which usually cluster together (Casjens et al., 2005). According to this, when the neighbor-joining tree was elaborated four clusters were seen and the terminases of the UAB_Phi20, UAB_Phi78, and UAB_Phi87 bacteriophages grouped together with those of bacteriophages with similar enzymatic end-generating functions (Figure 7). In this sense, UAB_Phi87 large terminase clustered into the *Felixounalikevirus* DTR group, and it was highly similar to terminases of *Salmonella* phages Felix O1 and FO1a, both with DTR in their chromosome ends (Whichard et al., 2010; Marti, 2013). In the same cluster were located terminases of phages wV8 and HY02 which infect *E. coli* and others from phages infecting *E. amylovora* or *Citrobacter*. UAB_Phi78 large terminase clustered together with that of *Salmonella* phage SP6 and *Lelliottia* phage phD2B which is a *Sp6likevirus* genus with a short DTR of 262 pb (Nowicki et al., 2014). As it was expected, the UAB_Phi20 large terminase clustered into the *P22likevirus* headfull group which included bacteriophages of *P22likevirus genus* as P22, ST64T or ST160. Thus, and as it has been pointed out (Casjens et al., 2005), the structure of virion DNA ends can be accurately predicted for phages although there is no previous experimental evidences, if their putative terminase amino acid sequence falls convincingly within one of those robust groups.

## Conclusions

Phage therapy is becoming an alternative or additional strategy to actual treatments of bacterial infections that can also help to diminish the emergence of antibiotic-resistant bacteria with difficult treatment. The use of bacteriophages requires a detailed characterization of these viruses. In this study, the genomes of three virulent *Salmonella* specific bacteriophages (UAB_Phi20, UAB_Phi78, and UAB_Phi87) were characterized in depth by functional genomic tools and their chromosomal ends were also determined. Detailed genome sequence analyses provided information about the three bacteriophages studied do not encode known virulence-associated or antibiotic resistance genes. The bacteriophages UAB_Phi78 and UAB_Phi87 contain terminal direct repeats in their chromosome which were identified. The UAB_Phi20 bacteriophage has a chromosome with circularly permuted DTR and it did not give rise to stable lysogens probably due to its inability to synthesize the lytic cycle repressor. This is consistent with both the complete clearance of infected-*Salmonella* cultures and the production of typical clear plaques. Genomic data and the comparison of terminases allow us the assignment of UAB_Phi20, UAB_Phi78, and UAB_Phi87 to the *P22likeviruses genus, SP6likeviruses* genus, and *Felixounalikeviruses* genus, respectively. This confirms the assignation reported for these bacteriophages obtained by different methods (Grose and Casjens, 2014). All the data obtained contribute to a better understanding of the biology of these phages which is necessary for the development and the use of an efficient cocktail with commercial applications in bacteriophage therapy as it has been showed (Bardina et al., 2012; Spricigo et al., 2013; Colom et al., 2015). The success of this cocktail could be attributed to the combined characteristics of the phages as their wide host-range, the different lytic cycles, and other particularities described in this study. To our knowledge, there are some reports about the use of bacteriophages closest to those studied by us but mainly in food (e.g., Whichard et al., 2003; Zinno et al., 2014), and only few in animals (e.g., Hurley et al., 2008) although with uneven results.

## Author contributions

CB and DS isolated and annotated the sequences the genomes of the three bacteriophages. JC carried out the final annotation, the characterization of the packaging process of the three bacteriophages, and the analysis of terminases. MS and JO participated in the characterization of the packaging process. PC and ML participated in the design and coordination of the study and in drafting the manuscript. All authors read and approved the final version of the manuscript.

### Conflict of interest statement

The authors declare that the research was conducted in the absence of any commercial or financial relationships that could be construed as a potential conflict of interest.
