# Supplementary material for: Genomics of Three New Bacteriophages Useful in the Biocontrol of Salmonella
Source: Front Microbiol. 2016 Apr 20;7:545. doi: 10.3389/fmicb.2016.00545 (PMC4837284; doi:10.3389/fmicb.2016.00545)
Supplement: Supplementary file 1 [file Table1.PDF]

Tabla S1. Putative promoters of phages UAB_Phi20, UAB_Phi78, and UAB_Phi87.

Phage	Position	-35	-10
UAB_Phi20	933	GTGATA	AGCTAATTT
	7,108	CTGCAG	GCTTAAAAT
	7,633	TTTAAT	CGTTAAAGA
	13,210	TTGCCA	AGACAAAAT
	18,214	CTGAAA	TGCTCTAAT
	28,905	TTGGCG	TCCTTTATT
	30,455	TTGATC	ATGTATAAT
	30,471	TTGATC	AATTAACAT
	31,952	TTGATG	TCTCAAGAT
	35,834	TTGAAA	CTTAAAAAT
	37,641	TTTTCA	AGTGAGTAT
	38,509	TTGTCG	GGTCACATT
	38,901	TTCACA	TTGTATCAA
UAB_Phi78	493	TTGGTC	TCTTATTCT
	799	TTGCTT	AGGTGAAGT
	1,246	TTGGTA	AACTATGAT
	1,663	TTGACA	GGTTACATT
	2,213	CTGAAA	CGCTAAAAT
	3,451	TTGACT	AAGTAAGAT
	6,823	ATGAGC	TCGTAAATT
	9,037	ATGATT	TGCCATGCT
	9,551	AGCACA	CGGTATCAT
	10,054	TTGTCT	CACTATCAT
	14,661	CTAACG	TGGTATCCT
	15,219	TTTAAA	AGCTATTAT
	16,320	TCGGCA	TGCTAGAAT
	18,275	TTGAAG	AGGTAAGAA
	19,367	TTGAGG	TGATATGCT
	20,049	TTAATG	ATGTATAAT
	23,132	TTCACA	AGGTAAGGT
	25,242	TCGCCT	ACTTAAACT
	27,601	CCGACA	TGGTAGAAT
	36,633	TTGCTA	AGGTAAAGT
	38,690	TGTACA	TACTATACT
	39,203	TTGAGA	CTGTATATT
	39,589	CTGACA	GCTTACACG
	39,890	TTACCA	CCGTAGGCT
	40,193	TACAAT	ATGTATCAT
	42,010	TTCACG	GGTTATCAT
UAB_Phi87	1076	TTGGAA	AGTTCAAAT
	3,743	TTAATA	GTTTTTTCT
	5,726	TTGACA	CTCTAAAGT
	9,709	TTGACA	GGTTATTAT
	11,931	TTAAAA	TTATATTAT
	12,664	TTGTAT	TGGTATCAT
	13,430	TTGTAG	AGGTATAAT

Tabla S1. Continued

Phage	Position	-35	-10
UAB_Phi87	13,878	TTATCA	ACTTAAAAT
	15,387	TTGTTA	TAACAAAAT
	16,549	TTGACG	CTGTAAATT
	18,037	TCGCAG	TTTTATAAT
	18,632	TTTACA	TGATATCTT
	18,936	TTGATT	AAGTAAATT
	20,506	TTTACA	TGGTAGGAT
	21,973	TTCATT	TTTTAAAAT
	23,707	TTGAAG	GTGTCTGAT
	24,013	TTGATA	GGTAAAGAT
	25,023	TTGCTA	GGTTAAGAT
	26,316	TTTACA	TCATATAAT
	27,984	TTGACA	CCTTAGAAT
	30,427	TTGTTG	TGGTAAAAT
	30,949	TTGACA	GTATATGAT
	33,726	TTACCA	CTGTAAAAT
	34,052	ATGAAG	ATCAAAAAT
	35,766	TTGTCA	GAGTAAAAC
	37,376	TTTCCA	CTTTAGAAT
	39,727	ATGCCA	GTTCATCAT
	43,581	GTGATA	TGGCAGAAT
	55,692	TTGAGA	ATTGACAAT
	55,845	TTTAAG	CACTACAAT
	56,135	TTTAAA	AGGTAGACT
	58,424	TTGACA	TTTTATACT
	60,180	TTCCTA	TGGTATTCT
	60,953	TTGAAA	CTGTATGAT
	62,577	TTGACT	TGTTAATAT
	72,038	TTGATG	TACTAAGAT
	74,285	TTGCTA	ATTTATTAT
	78,706	TTGAAG	TGCCATACT
	81,939	TTGTCA	TGATATGAT
	83,928	TTTCAA	AGTTAAATT
	85,791	TAGACA	AGTTATACT
	86,901	TTCAAA	ATTTATAGT
