# Supplementary material for: Genomics of Three New Bacteriophages Useful in the Biocontrol of Salmonella
Source: Front Microbiol. 2016 Apr 20;7:545. doi: 10.3389/fmicb.2016.00545 (PMC4837284; doi:10.3389/fmicb.2016.00545)
Supplement: Supplementary file 2 [file Table2.PDF]

Table S2. Location of the tRNAs in the UAB_Phi87 genome.

tRNA	tRNA beginning	tRNA end	Aminoacide	Anticodon	Codon	Cove Score
1	1100	1025	Arg	ACG	CGT	63.45
2	1183	1106	Leu	CAA	TTG	43.76
3	1259	1185	Val	TAC	GTA	51.84
4	1430	1355	Thr	TGT	ACA	59.75
5	1512	1438	Gly	TCC	GGA	56.5
6	1594	1519	Ala	TGC	GCA	46.25
7	1676	1601	Lys	CTT	AAG	59.65
8	1761	1684	Leu	TAG	CTA	48.98
9	2422	2346	Arg	TCT	AGA	74.9
10	2834	2759	Ile	GAT	ATC	68.23
11	2992	2917	Lys	TTT	AAA	60.2
12	3527	3451	Asp	GTC	GAC	68.38
13	3621	3534	Pseudo (Tyr)	GTA	TAC	39.92
14	3861	3785	Met	CAT	ATG	28.9
15	4030	3953	Glu	TTC	GAA	63.13
16	4114	4038	Pro	TGG	GGA	68.25
17	85986	85911	Cys	GCA	ACG	50.36
18	86083	85991	Pseudo (Ala)	GCT	AGC	41.09
19	86911	86836	Phe	GAA	TTC	43.77
20	86993	86918	Pseudo (His)	GTG	CAC	42.31
21	87100	87025	Gln	CTG	CAG	50.21
22	87184	87106	Leu	TAA	TTA	57.88
23	87262	87187	Gln	TTG	CAA	47.31
