# Supplementary material for: Genomics of Three New Bacteriophages Useful in the Biocontrol of Salmonella
Source: Front Microbiol. 2016 Apr 20;7:545. doi: 10.3389/fmicb.2016.00545 (PMC4837284; doi:10.3389/fmicb.2016.00545)
Supplement: Supplementary file 3 [file DataSheet1.PDF]

Supplementary Material

Genetics and genomics of three new bacteriophages useful in the biocontrol of *Salmonella*

Carlota Bardina†, Joan Colom†, Denis Augusto Spricigo†, Jennifer Otero, Miquel Sánchez-Osuna, Pilar Cortés [*], Montserrat Llagostera.

*** Correspondence:** mariapilar.cortes@uab.cat

1 Supplementary Figures and Tables

Supplemental material includes Table S1 and Table S2.

Moreover, it is also included one supplementary figure: Figure S1.

1.1 Supplementary Figures

Figure S1. Sequencing of the chromosomal ends of bacteriophages UAB_Phi78 (A) and UAB_Phi87 (B). The sequences of the short direct terminal repeats identified in both chromosomes are shown.
