# Supplementary material for: Genomics of Three New Bacteriophages Useful in the Biocontrol of Salmonella
Source: Front Microbiol. 2016 Apr 20;7:545. doi: 10.3389/fmicb.2016.00545 (PMC4837284; doi:10.3389/fmicb.2016.00545)
Supplement: Supplementary file 4 [file Image1.PDF]

A

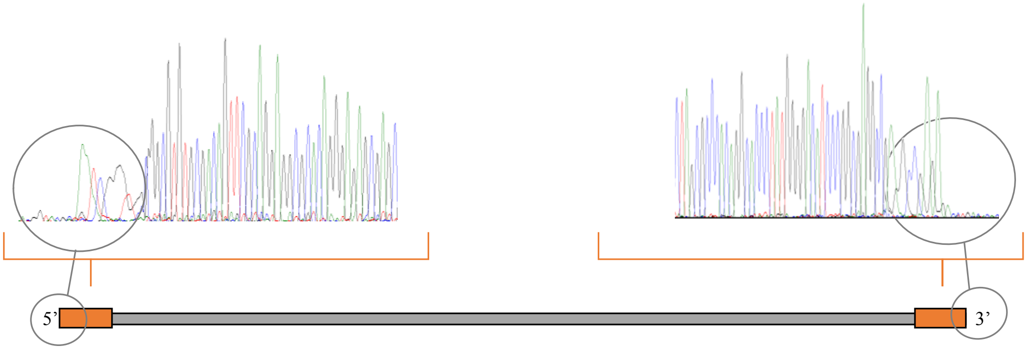

Direct terminal repeat of UAB_Phi78

5’-
TAGGCGTGTGTCAGGTCTCTCGGCCTCGGCCTCGCCGGGATGTCCCC
ATAGGGTGCCTGTGGGCGCTAGGGCGGCCTGAGAAGGCCTGAGAGA
GTCGCTTAGTGTGGGCCAAAGGGAGACCGGAGGCCGACCGAGAGCG
AGCGAGAGGGACACGCGGAGGACGCTTGACAGCGTGTGCGG-3’

B

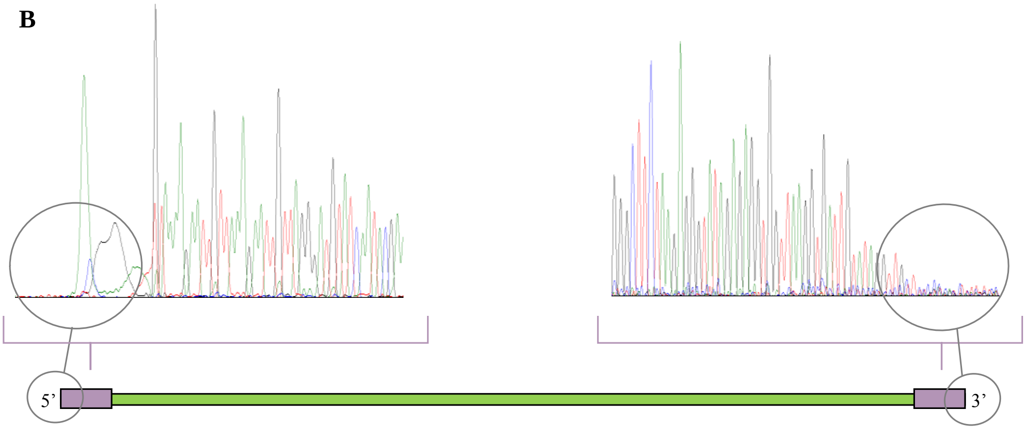

Direct terminal repeat of UAB_Phi87

5’-
AACATTTTCTTAACAATTTCTTAACAATCCCTACATAGTTATGCTTTTAAGGCACTT
GTTAGCAACTTGTTAGCCCCTTGTTAAATCTCTGTTAAGACAACGTAAAAGCCTTGT
AAGAGTCTTTGACATAGCCTTAAAAGCTCCTGTAAAGCTCTATAAAAAGGTGTCTG
TACAGTTGGGGTGTAACTACTAGGGAGAGGCTTAAAAGGGCTTACAAAGAGCTAT
AGAAAGCTTTTAAAAGGTATGTTTAAGGAATCACTAAGAAAGGAAAGGGAACGAT
AAGAAAAGGGATAGTGACCATGTTAATAGCTTGTTAAAAAGATTAGACACTTGTTA
GGAACTTGTTAATTATCTTAACAAAATAACTCTTAAAAATTAACTAATTGGTATTGT
TACCAATCCCTGTTAGCTCTTGTTAGTCATCTGTTAAGAGACTATAAAGACCATGTA
TCGACTGTTAAGAACTTGTTACAGCCTTGTTAGTTCTGTTAAGAATCTGTTAAGACA
TCTGAAGAGATGTTAAGAGCTTGAGAGAGTTGTTAAGAGATTGTTAATGATATTTT
AAAATTCTGCAAAGGGGATGTTACAGGGATGTTAAGGGCTACCAA-3’

Figure S1. Figure S1. Sequencing of the chromosomal ends of bacteriophages UAB_Phi78 (A) and UAB_Phi87 (B). The sequences of the short direct terminal repeats identified in both chromosomes are shown.
